# Shedding light on cashmere goat hair follicle biology: from morphology analyses to transcriptomic landascape

**DOI:** 10.1186/s12864-020-06870-x

**Published:** 2020-07-02

**Authors:** Cristina Nocelli, Katia Cappelli, Stefano Capomaccio, Luisa Pascucci, Francesca Mercati, Irene Pazzaglia, Samanta Mecocci, Marco Antonini, Carlo Renieri

**Affiliations:** 1 School of Pharmacy, University of Camerino, Via Gentile III da Varano, 62032 Camerino, Italy; 2 Department of Veterinary Medicine, University of Perugia, Via San Costanzo 4, 06126 Perugia, Italy; 3 Istituto Zooprofilattico Sperimentale dell’Umbria e delle Marche, Via Salvemini 1, 06126 Perugia, Italy; 4 Italian National Agency for New Technology, Energy and Sustainable Economic Development, ENEA CR Casaccia—SSPT BIOAG Probio, S.M. di Galeria, 00123 Rome, Italy

**Keywords:** Differentially expressed genes, Hair follicle cycle, RNAseq, Keratin 4

## Abstract

**Background:**

Cashmere goat is known for its precious undercoat. Being photoperiod-dictated, cashmere growth has been studied focusing mainly on hair follicle cycle phases (anagen, catagen and telogen). An accurate molecular knowledge of the goat hair follicle cycle, disentangling gene expression changes during phases and recognizing timing boundaries, could be useful to improve cashmere goat management and ultimately cashmere production.

**Results:**

To better describe goat’s hair follicle transcriptome we applied RNA-sequencing to isolated hair follicles from five Italian cashmere goats, during the anagen and catagen phase, identifying total of 214 differentially expressed genes (DEGs): 97 were up-regulated while 117 were down-regulated in catagen with respect to anagen. Gene Ontology and pathway analysis were performed. We detected 144 significant pathways spanning from estrogen, pluripotency of stem cells, thermogenesis and fatty acid metabolism that were strongly expressed during the hair follicle phases analysed. Finally, we validated promising DEGs by RT-qPCR in the same set of samples as well as in hair follicles and entire skin biopsies of another cashmere goats cohort accounting for early anagen, anagen, early catagen, and catagen phases.

**Conclusions:**

As in the isolated hair follicles, some target genes were homogenously modulated during the four hair follicle phases. Ceruloplasmin (*CP*) and Keratin 4 (*K4*), confirmed their clear cut expression between growing and resting phase. In fact, *K4* was almost absent in catagen phases while *CP* was barely expressed in anagen phases. In particular, the strong expression of *K4* in early anagen makes it an eligible marker to track the beginning of a new hair cycle, and therefore defining the optimum time for cashmere harvesting.

## Background

Mammalian species produce hair as protection against environmental factors. Many mammals from the temperate zone, modify seasonally their insulating capability in order to face temperature changes during the winter [1]. There are two kinds of coats: an uppercoat formed by guard hair for “physical protection”, being waterproof thanks to sebaceous glands secretion, and a down coat, with superior thermal insulation capability due to the air trapped within the coat [2]. The Cashmere goat is a double coated mammal [3], and its luxury underhair, the cashmere, is made by the secondary hair follicles (SHFs). SHFs are usually located in clusters of 6 to 15 and, for each group, [4] there are 1–3 primary hair follicles (PHFs) as a guard hair. The photoperiod is a relevant factor for seasonal coat change [5] and SHFs cells mitotic activity remains high from the summer to winter solstice before decreasing. This growing time span is recognized as the anagen phase of the hair cycle. With photoperiod increasing, the SHFs go towards a resting phase known as catagen, that ends with telogen (usually from February to March), where hair is easily dislodged and cashmere is harvested, usually by combing. Then, a new regenerative hair cycle is ready to begin.

The reshape of a new hair follicle (HF) from follicular keratinocytes is guided mainly from the dermal papilla cells (DPCs) that manage information generated by local and systemic hormons and molecules to promote hair growth [6]. These information can induce proliferation in noumerous populations of HF stem cells (HFSCs) dipped in the skin environment [7]. Some of these molecular signals are well known especially in single-coated mammals, such as mice and humans. The role of Sonic Hedgehog, WNTs and β catenin as promoters of the hair growth are largely outlined, as the involvement of BMPs pathways in the regression phase of the hair cycle [8, 9]. Lately, the principal canonical pathways of the HF cycle were assessed also in cashmere goats [10, 11]. Particularly, a recent works identified a strong signature of selection involved in cashmere production traits [12, 13], confirming the role of some molecules engaged in the generation and regeneration of hair, such as *Lhx2*, implicated in the SHFs development [14], *Fgf5,* whose disruption in cashmere goats led to more secondary hair follicles and longer fibers [15] and the transcription factor *Hoxc8*, whose hypermethylation status of exon1 is correlated with shorter fleece length on cashmere goats [16]. However, in double-coated animals, some other uncommon pathways could be involved in the undercoat growth.

Precise knowledge of genes and pathways involved in the HF cycle, and therefore the fine evaluation of molecules involved in the active growth and in the regressive phases of the fiber, can be used to plan the most favorable harvest time and may improve cashmere yield. Different kinds of gene expression analysis methods can be used (e.g., RT-qPCR, microarray, sequencing), and they differ in terms of robustness, throughput, accuracy, sensitivity, dynamic range, cost and complexity. With RNA-seq performed by Next-generetion sequencing (NGS) it is possible to provide accurate gene expression profiles of sequenced transcripts, detecting both novel and known mRNAs, performing a relative quantification of mRNA transcripts present at a low abundance. This is possible also for mammals for which limited genomic resources are available. Althought cashmere goats are reared mainly in Asia, mostly in China, Italy imports the largest amount of cashmere in the world for prestigious fashion industry brands. Local production is confined to small flock that yet preserve seasonal patterns. In our study an RNA-sequencing approach were used for reveals a picture of HFs transcriptome during follicle phases in Italian Cashmere goat population.

## Results

### Morphological analysis of isolated hair follicles and histomorphological evaluations

**Fig. 1:**
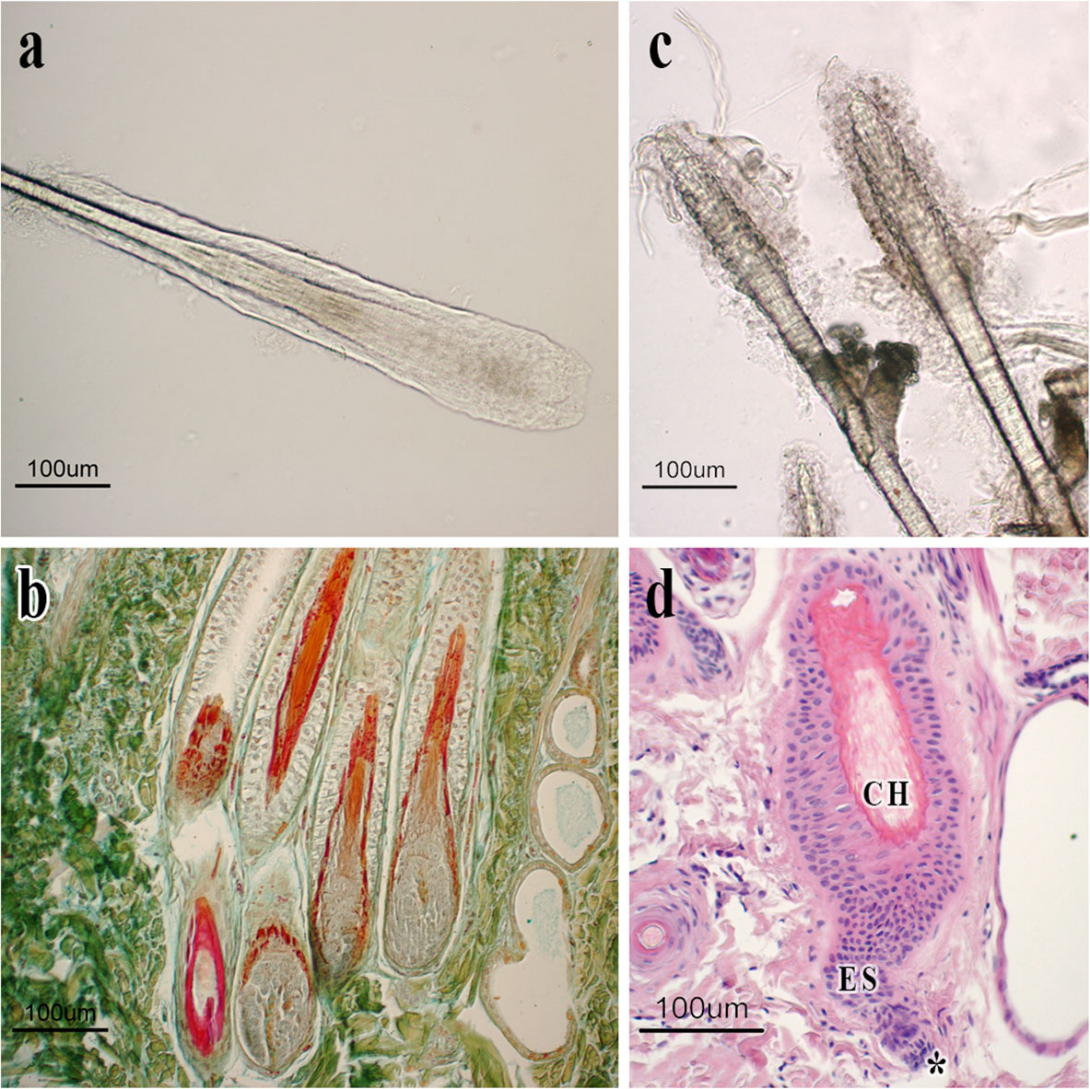
**a** Isolated HF in anagen phase. A hair enclosed by the epithelial sheath is shown. The bulb is completely developed and shows a round shape. **b** Histological section showing a group of secondary HFs in the anagen phase. The bulbs and soprabulbar regions are shown. Floxin B/Orange G/Alcian blue staining. **c** Isolated HFs in regressive phase. The bulbs are missing; hair is short and show a characteristic club shape. Sebaceus glands close to club hair can be observed. **d** An HF in catagen-telogen transition phase. Typical morphological features of this phases are shown. A little and extruded dermal papilla (*); the epithelial strand (ES); the club hair (CH). Haematoxylin-Eosin staining

Thanks to histomorphological evaluations, the SHFs in the anagen phase are easily distinguishable for the typical dilated and rounded morphology of the bulbs (Fig. 1a and b), the dermal papilla surrounded by hair matrix and the presence of inner root sheat (Fig. 1b). SHFs in catagen phase showed instead the peculiar club hair (Fig. 1c and d), a trichilemmal keratinization and a little dermal papilla below the secondary hair germ confirming their proximity to telogen phase (Fig. 1d).

### RNA sequencing data analysis

**Fig. 2:**
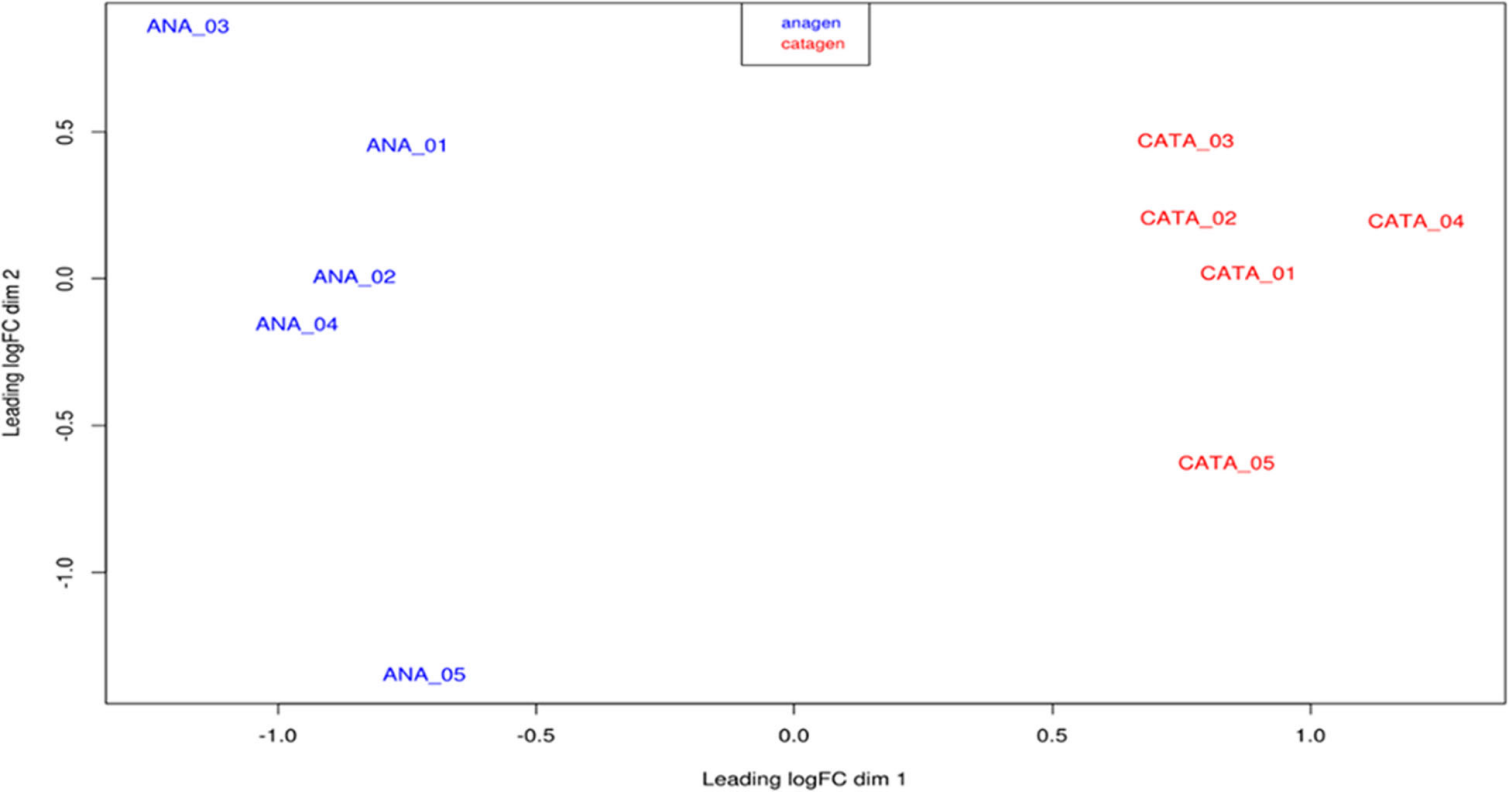
MDS output processed with EdgeR. Anagen samples are clearly separated from catagen samples

The transcriptome analysis of isolated HFs in anagen and catagen of 5 yearling cashmere was performed. The experiment produced a total of 860 million reads, 86 million reads per sample. RNA sequencing provided high quality reads with good similarity among samples. Multidimensional scaling analysis (MDS) of fold-change differences in gene expression shows relationships between samples in each group and a good separation between anagen and catagen (Fig. 2). Quality control and trimming procedures retained the vast majority of the sequences produced (from 87 to 97% of the total) from an average of 43,337,566 to 39,728,735 reads. Alignment was successful for 79 to 89% of the cleaned reads, and a good proportion of unique alignments was observed with an output of average 34,071,742 mapped reads. Only these sequences were used for the differential gene expression assessment to avoid introducing bias through multi-mapper assignment uncertainty. An overview of trimming and mapping data is shown in the Additional file 1.

### Differentially expressed genes

**Fig. 3:**
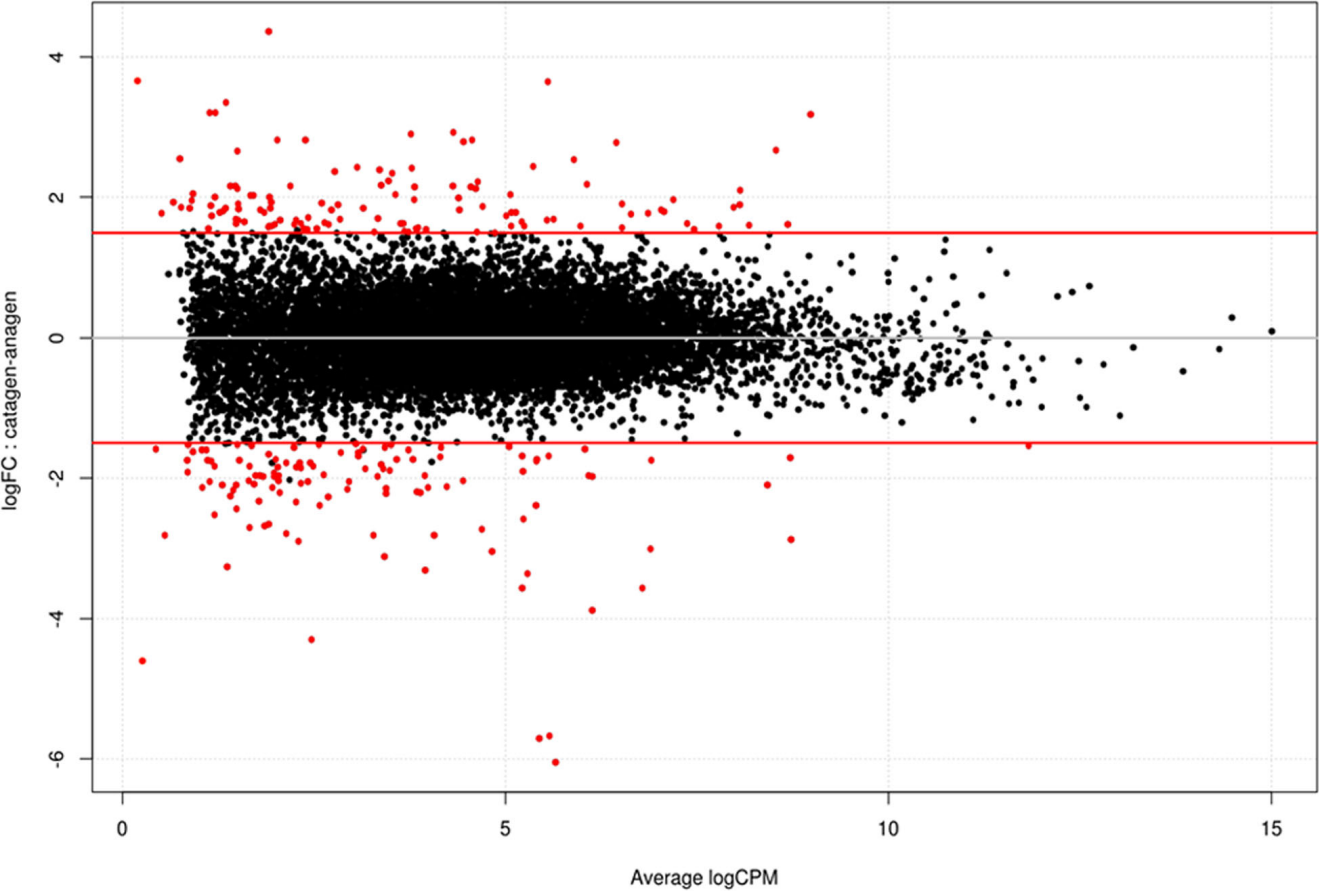
Smear plot of the total analyzed genes. Red spots denote differential expressed genes

After a statistical analysis with edgeR using a data set of 12,486 filtered genes, we found 214 differentially expressed genes (DEGs) in the isolated HFs, with a significance of *q* < 0.05 and an absolute fold change (logFC) greater than 1.5. Using these filters and setting anagen phase as a control, 97 genes results up-regulated (logFC > + 1.5), whereas 117 genes were down-regulated (logFC < − 1.5) with respect to anagen (Fig. 3). Full results are shown in the Additional file 2.

### Gene functional analysis

**Fig. 4:**
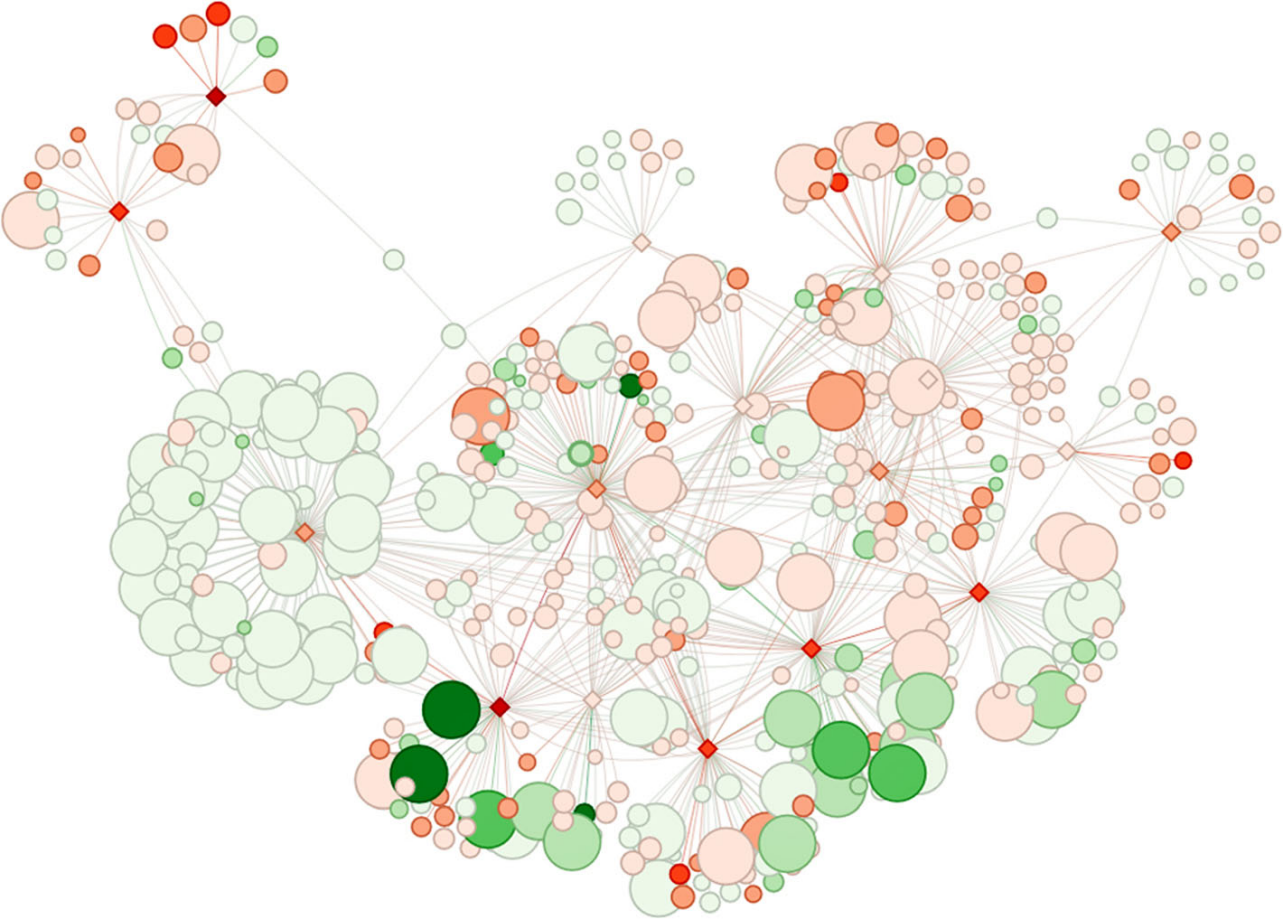
PIA gives a graphical output to facilitate the understanding of the pathway relationship amongst genes. The intensity of the color of the red diamonds give an idea of the expression level of the pathway. Furthermore, fixing anagen as reference, the color intensity of the red balloon highlights the up-regulation in catagen. While the intensity of the green balloon gives information about the down-regulation in catagen. A more detailed resolution is visible at the link https://github.com/CristinaNocelli/ghf_enrichment/blob/master/README.md

After the annotation of modulated transcript list through BioMart, gene names retrieved were used to perform an enrichment analysis with BiNGO, a Cytoscape app. However, due to the limitation of *Capra hircus* gene annotation, we evaluate them also in the vocabularies of *Bos Taurus*. Furthermore, we coupled the GO analysis approach with a more general one using a new pathway analysis tool. We used as input our differential expressed genes list coupled with selected pathways related to HF growth. After filtering and enrichment procedure, the tool generated a network with the most significant pathways (FDR < 0.05, after Benjamin-Hochberg correction). With the highly interconnected network identified, we focused on enriched pathways and biological processes (FDR < 0.05): analysis pointed out 144 statistically significant pathways. Among them, some are peculiar of the HF cycle activities such as “thermogenesis”, “circadian rhythm” and “regulation of pluripotency of stem cells”. Results are provided with an interactive graphical visualization for nested exploration of pathways and differentially expressed genes (Fig. 4). Additional file 3 contains all enriched pathways and for a more detailed visualization follow this link: https://github.com/CristinaNocelli/ghf_enrichment/blob/master/README.md

### qRT PCR

**Fig. 5:**
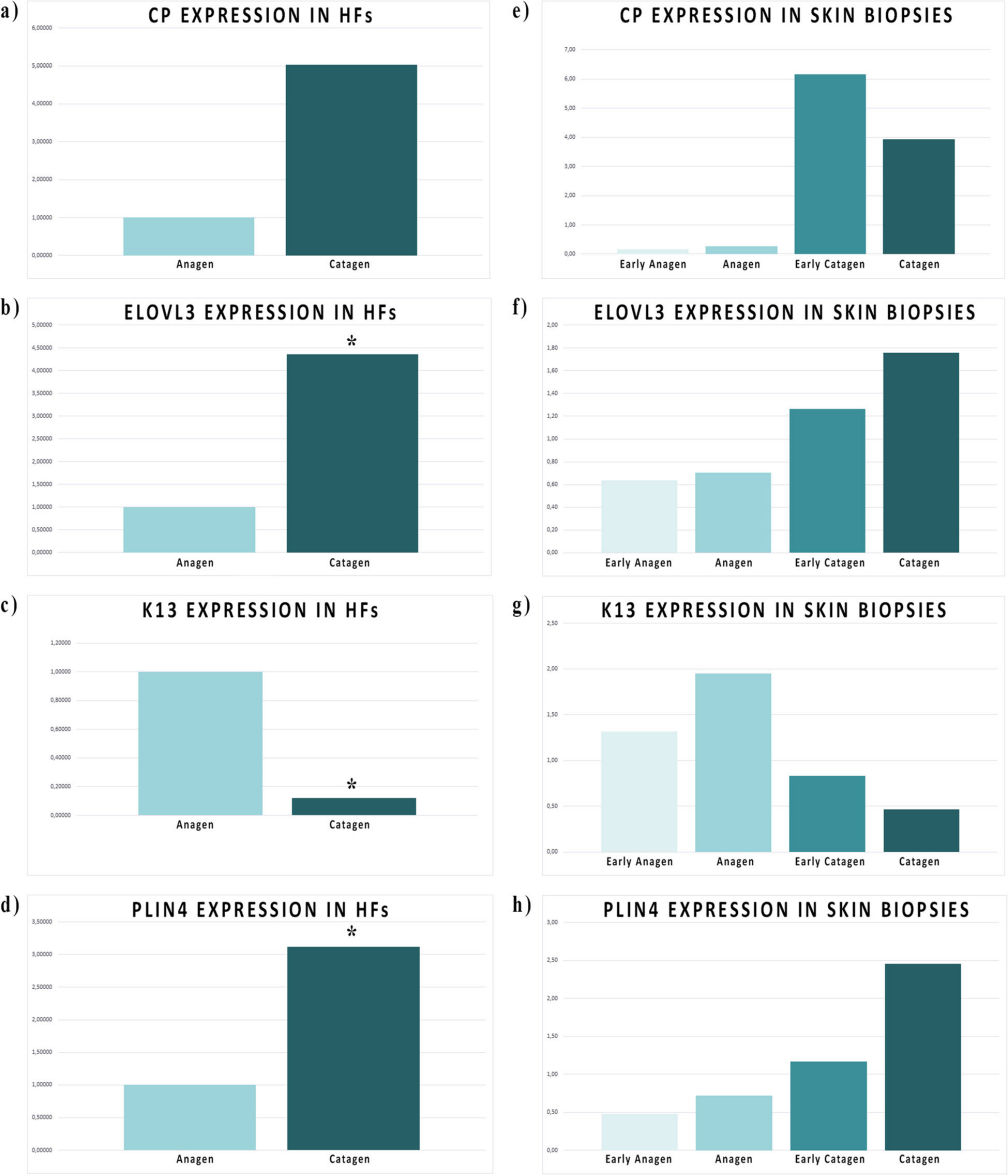
Candidate gene expression in HFs and in skin biopsies. The bar chart shows the expression between anagen and catagen in the HFs (**a**, **b**, **c** and **d**). Light blue bars focus on the anagen phase, while blue bars point out the catagen phase. For the HFs, significant genes evaluated through t-test (*P* < 0.05) are marked with the symbol (*). While in the skin biopsies (**e**, **f**, **g** and **h**) is possible to evaluate the same genes during early anagen, anagen, early catagen and catagen phases. Regarding skin biopsies, the significance of the expression level (*P* <0.005) is evaluated as ANOVA test and is visible in the Additional file 5

**Fig. 6:**
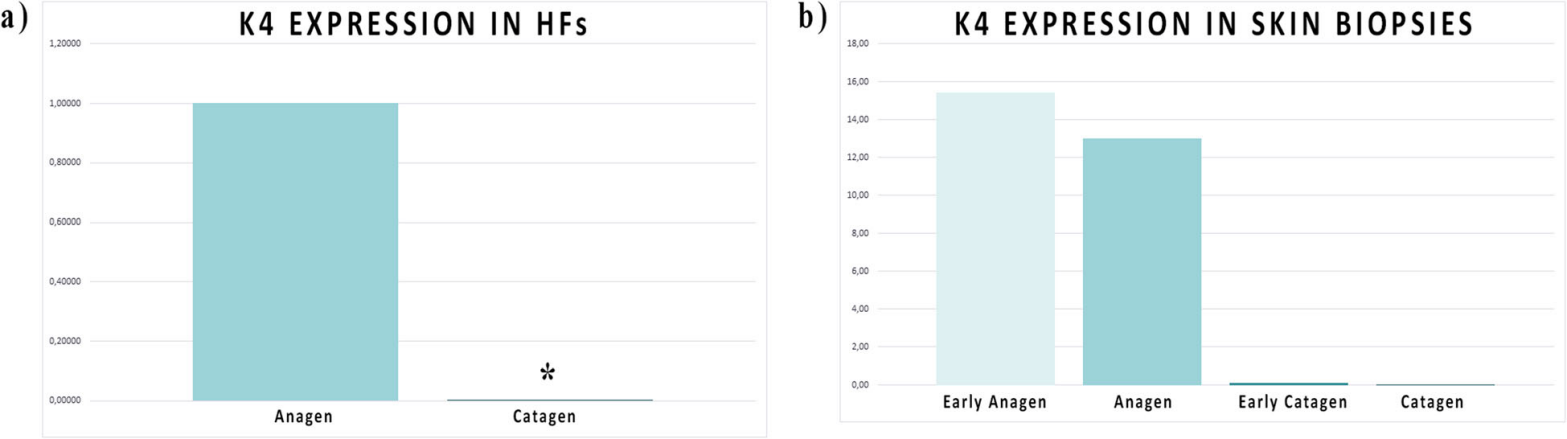
A detatiled picture of the *K4* strong expression in HFs (**a**) and in skin biopsies (**b**) during growing phases (early anagen and anagen) and not less, the really low expression during regressing phases (early catagen and catagen)

A selection of DEGs, have been evaluated by RT-qPCR for validation in the isolated HFs (two phases, anagen and catagen, Fig. 5a, b, c and d; Fig. 6a) and to confirm modulation in whole skin biopsies (four phases, early anagen, anagen, early catagen and catagen, Fig. 5e, f, g and h; Fig. 6b). The selection has been done considering different parameters such as log fold change (logFC) and log counts per million (logCPM) for each gene, with special attention for the functional correlation of these genes to HFs of other species retrieved by literature and/or KEGG database.

Keratin 4 *(K4)* and Keratin 13 *(K13)* are strongly differentially expressed in isolated HFs: in particular, *K4* expression ratio level highlights extreme up-regulation in anagen. As expected, *Plin4*, a member of the perilipin family, involved in coat intracellular lipid storage droplets, is fairly expressed during the cold season. The same pattern is observed for Elongation of Very Long Chain Fatty Acids Protein 3 (*Elovl3*) also named Cold-Inducible Glycoprotein, underlining how HFs could be influenced by environmental temperature. Ceruloplasmin (*CP)* is moderately expressed in HFs but the great individual variability negatively effects statistical significance. In anagen this gene appear to be expressed below our detection limit.

## Discussion

**Fig. 7:**
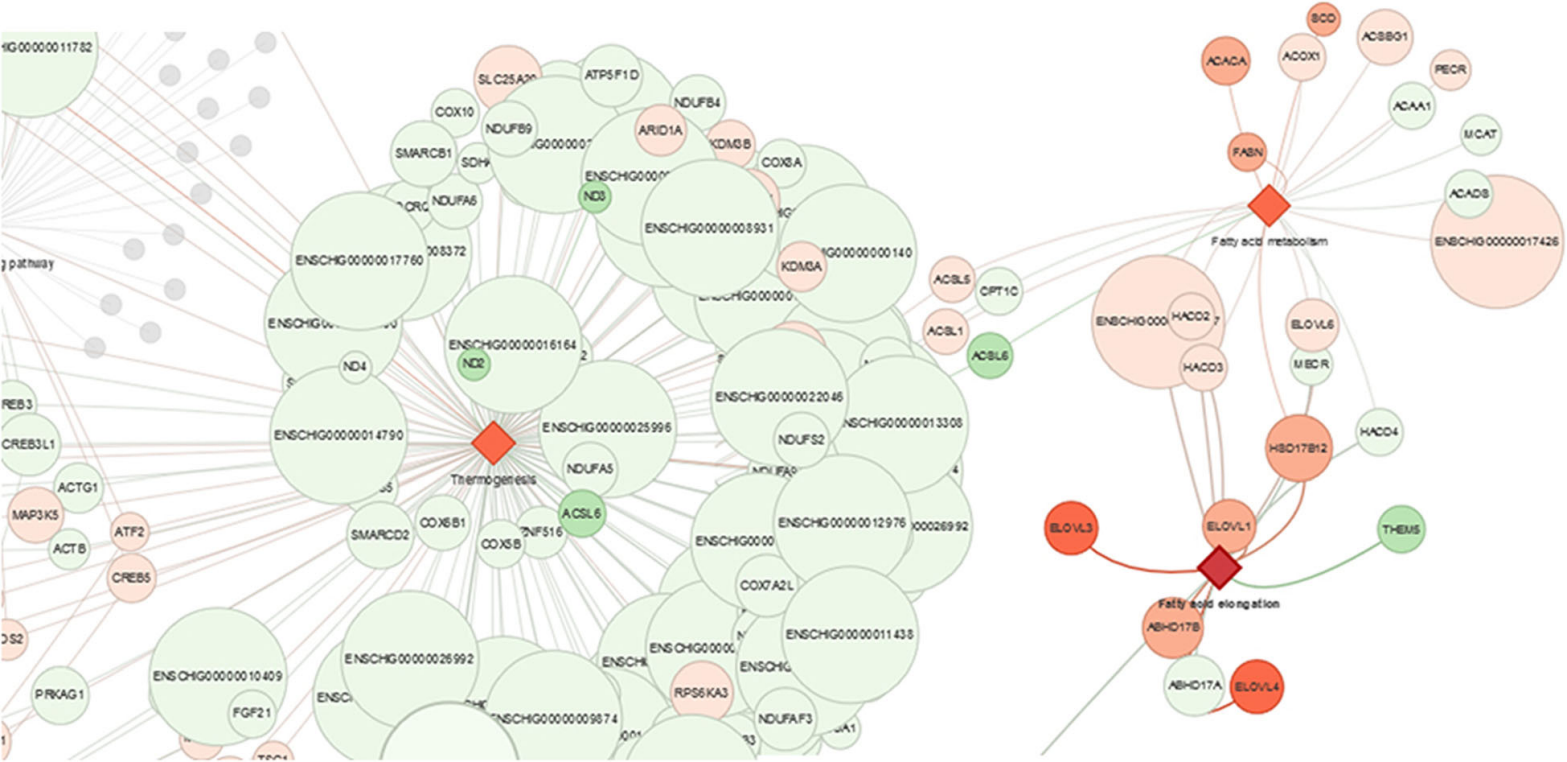
Some genes such as molecules involved in Acyl-CoA Synthetase are linked with both thermogenesis and its related fatty acid metabolism and fatty acid elongation pathways. The most of other genes of the thermogenesis are up-regulated in catagen, however some of them do not have a great differential expression among the HF phases

**Fig. 8:**
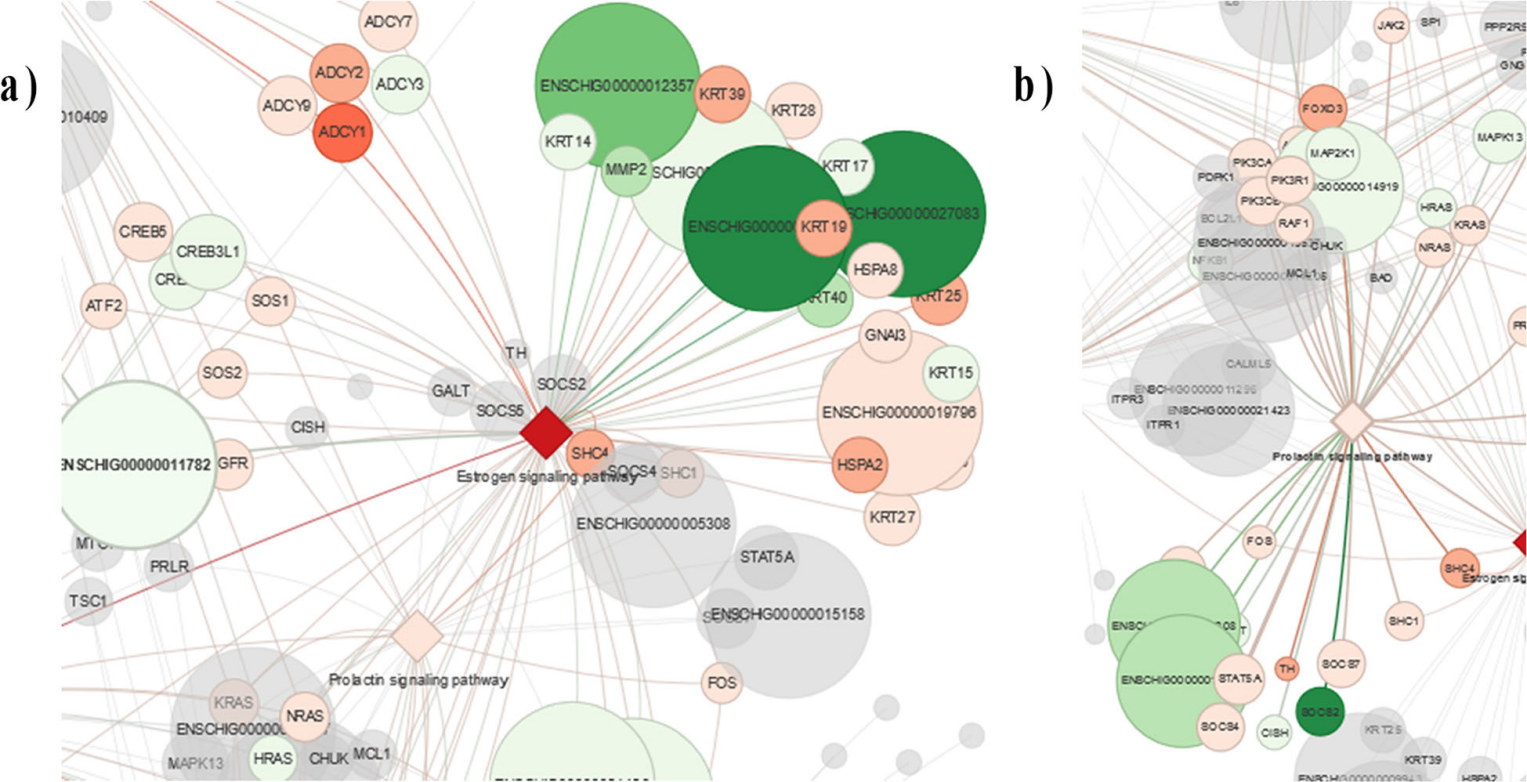
The estrogen signalling pathway (**a**) is solid active instead, prolactin pathway (**b**) results are not in the group of the strongest expression level

In this study, similarly to Su et al. 2018 [17], isolated HFs have been chosen as an alternative to skin biopsies to explore hair growth cycle molecular signature in cashmere goats. Compared to skin biopsy, that represents a much more complex cellular substrate, the isolated HF should enhance the signals from hair follicle stem cells and reduce “dilution” effects when comparing specific genes at different phases (catagen and anagen). With Pathway Interaction Analysis (PIA) [18], we evaluated some HF cycle canonical pathways such as the pluripotency of stem cells and circadian rhythm including some uncommon pathways. Interestingly, genes involved in thermogenesis and its related fatty acid metabolism and fatty acid elongation pathways are significantly enriched in catagen phase (Fig. 7). This finding underlines the major role of environmental temperature and points out the importance of fatty acid related pathways in the cashmere cycle especially during the cold winter season. Surprisingly, prolactin signalling pathway, known to stimulate hair shaft elongation in vitro in cashmere goats [19], is rather passive in our study. Conversely, the estrogen signalling pathway is strongly activated (Fig. 8). Some keratins (*Krt* or *K*) like *K23, K19, K39, K25, K28* are linked with this pathway and positively modulated in catagen, whereas *K13, K17, K15*, and *K40* are up-regulated during anagen. Either prolactin and estrogen are closely related and are subjected to melatonin direct and indirect effects. Despite the fact that prolactin levels increase following ovulation, leading to a seasonal moult [20], it seems by our evidences that genes in the estrogen pathway exceed the activity of the prolactin pathway and its cluster of genes. This suggests a direct role of the estrogen in the control of HF cycle, modulating the 105 genes annotated in this pathway. The adenylate cyclase1 (*Adcy1*) is the most up-regulated gene in estrogen pathway during catagen. Despite *Adcy1* is generally linked with thermogenesis pathway [13], it has been recently associated to hypertrichosis in mice. Although estrogen mediation is remarkable in the HF development, the impact of this pathway in the cashmere growth need to be further investigated. Interestingly, circadian rhythm is linked with thermogenesis pathway and cell cycle through molecules related to the regulation of cell energetic metabolism. However, from our data, the major part of genes from this cluster are not modulated during HF phases, as confirmed recently by Wu et al 2020 in goat skin [21]. Genes related to pluripotency of stem cells, mainly recruited during anagen, are also related to Wnt signalling, that has been shown to be a critical regulator of HF development and cycling [22]. For example *Bmpr1A*, a stem cell repressor [23], is linked with the pluripotency of stem cells pathway and is consistently expressed during catagen. Furthermore, genes involved in the pluripotency of stem cells are interactively linked to a series of canonical pathways, known to be implicated in the HFSCs fate, such as prolactin and estrogen signalling [20], Hippo signalling [22] and other HF pathways like TGF-β, and Sonic Hedgehog [8]. The strong expression of pluripotency of stem cells pathways, represented by genes expressed both during growth and regression, supports how stem cells cycle are deeply engaged in the cashmere regeneration. One of them, PI3K-AKT-mTOR pathway, is crucial in the coordination of cell survival, differentiation, proliferation, and development during organogenesis [24]. Moreover, PI3K-AKT-mTOR pathway mediates pathophysiological processes in several mammals including goat [25]. In our HFs, this pathway is connected, in addition to pluripotency of stem cells, with estrogen, cell cycle, and Notch signalling pathways, suggesting thath of PI3K-AKT-mTOR pathway is central in the cashmere growth. All these results point to the morphogenesis and the maintenance of new HFs, confirming that isolation of HFs is mandatory to better describe hair growth in cashmere goats. Concerning keratins, that are key structural components of HFs and hair, a *K4* expression has been detected. This is uncommon in HFs, even if described in other epithelia (i.e. oral mucosa [26]). The clear differential expression of *K4* during hair cycle phases is a promising biomarker that could be useful to detect the optimal time for cashmere combing, rather than others DEGs [27]. *K4* is not linked with any KEGG pathways in any species, while in humans is defined as an ortholog of *K13* [28], recently ascribed as biomarker of the dermal stem cells [29]. Both *K13* and *K4* seems to be found as a protein in the exosomes in human urine [30], but nothing is known about the role of *K4* as a signalling molecule. Curiously *K4* is mentioned in a human study regarding an eye pathology, the pterygium, an overgrowth of fibrovascular tissue from conjuntiva across the corneal surface associated with sunlight exposure [31]. Interestingly, the most abundant molecules expressed in the study of Jaworski and colleagues [31], besides *K4,* are *K13* and solute channels involved in mineral transport such as Aquaporin 3 (*AQP3*) as well as the *S100A* family, which are also implicated in our research. Recently *K4*, *AQPs* and the S100 cluster were inserted in the group of seasonal rhythm genes (SRGs) also in cashmere goat skin [21]. The last-mentioned S100 seems to be a marker for hair follicle stem cells in mice, both in the bulge and in secondary hair germ [21], while *AQP3* is a water channel protein involved in neonatal skin inflammation and eyes morphogenesis [21, 32]. These results in concert with Wu et al. 2020 [21] study in whole goat skin, revealed that some molecules, also in the isolated HFs, could be potentially photosensitive. Accordingly, some cells affected by those molecules could maintain the specific rhythm of the peripheral organs based on the photoperiod [21]. Thus, the HF as a microorgan could be light sensible. Moreover, the regeneration of new HF is identifiable with fibrovascular tissue proliferation and reveals how micronutrients switching is crucial during HF growth and in senescence phases. In this context *CP* acts as a metalloprotein binding most of the copper in plasma and exert its role in iron peroxidation. As recently remarked by Almohanna et al., 2019 [33], the association of hair loss in women and iron deficiency is particularly known in telogen effluvium. Despite not being expressly named in the mineral pathway, *CP* was recently included in the KEGG Porphyrin and chlorophyll metabolism, which is clearly linked to the Ferritin and Apotransferitin pathway. In humans, *CP* mutations cause a rare disorder named Menkes disease, also known as kinky hair disease, characterized by trichothiodystrophy and steely hair [34]. Intriguingly this gene is almost absent during the early anagen and anagen phases, but it is up-regulated during the early catagen and catagen phases. Since it is not expressed in a large amount it is not serviceable as a marker for the moment.

## Conclusion

In this study some useful markers have been identified to define the time boundaries of a new HF cycle in a relatively new population of Italian cashmere goats. The assessment was conducted over the entire follicular cycle, in addition, it was useful to confirm the modulation of cashmere production in these double coat animals through gene expression. Moreover, similarities with the DEGs output of the Chinese cashmere goats, indicate a relationship between these kinds of cashmere goat breeds. Through the use of adequate tools to exploring pathways, it has been possible to evaluate some unexpected links among interesting genes expressed in pathways arising HFs during anagen and catagen. This is particularly clear for those involved in environmental effects such as thermogenesis and fatty acid metabolism. In addition, the description of DEGs pointed to unsuspecting genes that can be related with HF cycle: such as *CP*, or a relative unpopular keratin into skin tissue as *K4* that makes it an eligible marker. Nevertheless further studies are necessary to identify the correct location into the HFs of this molecule.

## Methods

### Ethics approval and consent to participate

The cashmere goats samples used in this project are the same used in Pazzaglia et al. [35]. All the skin biopsies were collected during 2012–2013, before that the Italian Government transposes the directive on animal welfare (all goats sampled are still alive and in health).

### Samples collection

#### Skin biopsies collection

Skin biopsies were collected from twelve (1 year) Italian cashmere does by punch biopsy method (0.8 cm diameter) from the Chianti Cashmere goat farm of the DVM Nora Kravis, Azienda Agricola La Penisola, 53017 Radda in Chianti, Siena,Tuscany, Italy (43°29′43.2″N 11°23′08.4″E) [35]. Five of them were used for RNAseq and the other seven samples were destinated to RT-qPCR.

The sampling of the five unrelated does was performed in August (during anagen phase) and January (catagen phase). Biopsies have been collected from the lateral thoracic region of each goat. Before sampling each doe has been locally anesthetized with lidocaine, to avoid any sufferance to the animals. Then the hair was cut and skin was disinfected with povidone-iodine solution. To ease healing, each goat was treated with One VET spray (Endospin, Italy). Skin samples aimed to HFs isolation were preserved and rinsed, before being processed, in a solution of phosphate buffered saline (PBS) and Gibco™ Antibiotic-Antifungal (100X) (Thermo Fisher Scientific, Massachusetts, USA), used to prevent bacterial and fungal contamination.

The sampling of seven skin biopsies were collected by punch biopsy method (0.8 cm diameter) from June to February, respectively in different periods: June (early anagen), September (anagen), December (early catagen) and February (late catagen) as previously described in the Pazzaglia et al. experiment [35].

#### Isolation of hair follicles

Following the human HF culture protocol of Inoue and Yoshimura [36], the instruction of Ohyama and Kobayashi [37], in dog HF isolation, after rinsing, skin biopsies were placed into PBS in a petri dish into a sterile hood. Then subcutaneous fat was trimmed out using spring microscissors and forceps. Skin biopsies were sliced into strips. As the HFs were well anchored to the dermis, an incubation in a solution of PBS and dispase (5 U/ml, Stemcell Technologies TM, Canada) has been performed at 37 °C for 40–45 min, where skin strips were freely floated. Thereafter, all the hair were scraped off the skin using curved forceps and scalpel, in an anterior to posterior direction. HFs plucked off from dermis were collected into 2 ml tubes and pelleted by centrifugation. Then HFs have been preserved into PureZOL™ RNA Isolation Reagent (Bio-Rad, California, USA) at − 80 °C pending the following RNA extraction.

### Morphological analysis of isolated hair follicles

Isolated HFs were accurately evaluated to determine the stage of the HF cycle. Once separated, they were just placed on a slide preserved in PBS solution, observed under a photomicroscope (Nikon Eclipse E800, Nikon Corp., Tokyo, Japan) and pictured through image management system ACT1 (Nikon Corporation), a software for microscope camera.

### Histomorphological evaluation

To establish whether the collected follicles were in anagen, catagen or telogen, we performed a gross morphological analysis of the whole isolated HFs and histomorphological evaluations on paraffin embedded HFs. One-half of each biopsy sampled as above described was used for morphological evaluations. Specimens were quickly fixed in a 10% formaldehyde solution in PBS (0.1 M, pH 7.4) and processed until they were embedded in paraffin wax [38]. Histological sections were stained with Haematoxylin & Eosin, Sacpic method [39], and Floxin B/Orange G/Alcian blue [34], and then they were accurately analyzed under a photomicroscope (Nikon Eclipse E800, Nikon Corp., Tokyo, Japan) to determine the stage of the follicular cycle.

### RNA sequencing

#### RNA extraction from isolated hair follicles and library preparation

Hair follicles preserved into PureZOL™ were subjected to RNA extraction with Aurum™ Total RNA Fatty and Fibrous Tissue Kit (Bio-Rad), following the manufacturer’s instructions.

The quality and quantity of total RNA extracts were measured using NanoDropTM spectrophotometer (Thermo Fisher Scientific,). Furthermore, the microfluidic electrophoresis on the BioAnalyzer 2100 (Agilent Technologies, California, USA) revealed the integrity and suitability of the RNA samples for NGS library preparation. Libraries were prepared according to the Illumina TruSeq2 kit, using poly-A mRNA purification. A total of 10 RNA samples, 5 for anagen and 5 for telogen respectively, were sequenced on one lane of Illumina HiSeq 1500 platform generating 150 base-paired end reads, according to the TruSeq2 kit.

### Data analysis

#### Quality check and mapping

The quality of raw sequences was checked using FastQC. The adapter removal and trimming were done with Trimmomatic v.0.33 [40]. Afterward, high-quality reads were aligned using STAR v.2.4.0.1 [41], to the goat reference genome ARS1 (USDA ARS, August 2016) setting the other parameters to STAR default values. HTSeq v.0.6.1 was used to quantify the mapping of the reads at each gene locus based on genomic features annotated in NCBI genomes of goat ARS1.

#### Differentially expressed genes and gene ontology analyses

A matrix composed of 12.515 rows representing the ARS1 transcript annotation was imported into R. DEGs was assessed following best practices for the EdgeR package. A transcript was considered differentially expressed if the False Discovery Rate (FDR) adjusted *p*-value (*q*-value) was lower than 0.05 and absolute logFC was > + 1.5. Differentially expressed transcripts between anagen and catagen were annotated using BioMart, (http://www.ensembl.org/biomart/martview/), and corresponding gene names were used to perform Gene Ontology enrichment analysis. Using the Cytoscape 3.3.0 plugin BiNGO 2.44 (Biological Networks Gene Ontology) [42] and ClueGO 3.2.0, our differentially expressed genes list was utilized as the input for Cytoscape and each Ensembl Transcript ID was substituted by the corresponding EntrezGene ID. Due to the scarcity of the *Capra hircus* gene annotation, a Pathway Interaction Analysis (PIA) [18] procedure has been carried out. PIA is a tool that attempt to find a connection between genes using the interaction described in the KEGG database [43] starting from user-defined pathways of interest and the RNA-Seq results gene list.

### RT-qPCR experiment

Primers were designed using the Primer-BLAST [26] software (https://www.ncbi.nlm.nih.gov/tools/primer-blast/). Reference genes were chosen in accordance with the authors Bai et al. that in 2014 [10] selected and validated eight housekeeping genes in skin tissue of Liaoning Cashmere goat. For our study succinate dehydrogenase complex subunit A (SDHA) and Ubiquitin C (UBC) were chosen. Each pair of primers as designed on two different exons, as an additional precaution to avoid amplifying genomic areas that are possible contaminants. As in the most GenBank databases as NCBI, some genes of goats are predicted, primers were primarily validated with a mold tool [44] (http://unafold.rna.albany.edu/?q=mfold) to verify if the predicted amplicon folding form were acceptable without any unfunctional pairing as a hairpin. Specificity of amplification was confirmed by sequencing. For each pair of primers, a preliminary RT-qPCR was performed and efficiency (E) was assessed assigning slope values and correlation coefficients (R^2^). All details are displayed in Additional file 4. Amplifications were performed using SsoAdvanced Universal SYBR Green Supermix (Bio-Rad) from a 1:20 cDNA dilution derived from 400 ng of RNA purified as previously described. Amplification was carried out by the instrument CFX96 Touch Real-Time PCR (Bio-Rad), using a two-step amplification protocol with the initial denaturation at 95 °C for 3 min followed by 50 cycles of denaturation at 95 °C for 10 an annealing-extension temperature of 58 °C for 15 s. Each reaction was carried out in triplicate and the Ct values (threshold cycle) obtained from CFX Manager™ Software (Bio-Rad) were retained only when a standard deviation was lower than 0.2. The Melt Curve analysis was set up in a range from 56° to 95 °C, with temperature increments of 0.1 °C/sec. The output of the reactions was analyzed by qbase+, a software developed by Biogazelle for processing RT-qPCR data through ANOVA test (skin biopsy) or t-test for paired samples (HFs). Statistical significant value were corrected using the false discovery rate (FDR) through Benjamini–Hochberg procedure.

## Supplementary information

**Additional file 1.** Output of trimming and mapping.

**Additional file 2.** List of DEGs.

**Additional file 3.** Output of significant gene enrichment processed by PIA.

**Additional file 4.** Condition and efficiency of primers used for the RT-qPCR experiments.

**Additional file 5.***P*-value < <0.005 of a target genes evaluated in skin biopsies through ANOVA test.

## Data Availability

The datasets analysed during the current study are available in the additional files. In particular: -Additional file 1 shows trimming and mapping data output. -Additional file 2 display the list of DEGs. -Additional file 3 expose the list of significant gene enrichment processed by PIA. -Additional file 4 reveals the condition and efficiency of primers used for the RT-qPCR experiments. -Additional file 5 exhibit the *P*-value < 0.005 of a target genes evaluated in skin biopsies through ANOVA test. The graphical output generated with PIA is available at the github repository https://github.com/CristinaNocelli/ghf_enrichment/blob/master/README.md Furthermore sequences of cashmere goats analyzed can be found at NCBI BioSample database https://www.ncbi.nlm.nih.gov/biosample SAMN12350248, https://www.ncbi.nlm.nih.gov/biosample/?term=SAMN12350248 SAMN12350249, https://www.ncbi.nlm.nih.gov/biosample/?term=SAMN12350249 SAMN12350250, https://www.ncbi.nlm.nih.gov/biosample/?term=SAMN12350250 SAMN12350251, https://www.ncbi.nlm.nih.gov/biosample/?term=SAMN12350251 SAMN12350252, https://www.ncbi.nlm.nih.gov/biosample/?term=SAMN12350252 SAMN12350253, https://www.ncbi.nlm.nih.gov/biosample/?term=SAMN12350253 SAMN12350254, https://www.ncbi.nlm.nih.gov/biosample/?term=SAMN12350254 SAMN12350255, https://www.ncbi.nlm.nih.gov/biosample/?term=SAMN12350255 SAMN12350256, https://www.ncbi.nlm.nih.gov/biosample/?term=SAMN12350256 SAMN12350257. https://www.ncbi.nlm.nih.gov/biosample/?term=SAMN12350257 The accession numbers corresponding to the DEGs found in the isolated HFs can be found in Additional file 2 and were sourced from the ENSEMBL genome browser. The accession numbers corresponding to the genes that were amplified in the RT-qPCR experiments can be found in Additional file 4 and were sourced from the NCBI database.

## Abbreviations

DEGs: Differentially expressed genes
DPCs: Dermla papilla cells
FDR: False discovery rate
HF: Hair follicle
HFSCs: Hair follicle stem cells
logFC: Absolute fold change
logCPM: Counts per million
MDS: Multidimentional scaling analysis
NGS: Next generation sequencing
PHF: Primary hair follicle
PIA: Pathway interaction analysis
SHF: Secondary hair follicle
SRGs: Seasonal rhythm genes

## References

[1] Su R, Zhang W-G, Sharma R, Chang Z-L, Yin J, Li J-Q (2009). Characterization of BMP2 gene expression in embryonic and adult Inner Mongolia cashmere goat (Capra hircus) hair follicles. Can J Anim Sci.

[2] Johnson E, Orfanos CE, Montagna W, Stüttgen G (1981). Environmental influences on the hair follicle. Hair research.

[3] Allain D, Renieri C (2010). Genetics of fibre production and fleece characteristics in small ruminants, Angora rabbit and South American camelids. Animal.

[4] Ansari-Renani HR, Ebadi Z, Moradi S, Baghershah HR, Ansari-Renani MY, Ameli SH (2011). Determination of hair follicle characteristics, density and activity of Iranian cashmere goat breeds. Small Rumin Res.

[5] Allain D, Laker JP (1994). Hormonal control of fibre growth and shedding: proceedings of a European workshop held in Toulouse, France on the 13th–14th December, 1993. European Fine Fibre Network.

[6] Ma S, Wang Y, Zhou G, Ding Y, Yang Y, Wang X (2019). Synchronous profiling and analysis of mRNAs and ncRNAs in the dermal papilla cells from cashmere goats. BMC Genomics.

[7] Joost S, Zeisel A, Jacob T, Sun X, La Manno G, Lönnerberg P (2016). Single-Cell Transcriptomics Reveals that Differentiation and Spatial Signatures Shape Epidermal and Hair Follicle Heterogeneity. Cell Syst.

[8] Alonso L, Fuchs E (2006). The hair cycle. J Cell Sci.

[9] Korosec A, Lichtenberger BM, Marques AP, Pirraco RP, Cerqueira MT, Reis RL (2018). In vitro models to study hair follicle generation. Skin Tissue Models.

[10] Bai WL, Yin RH, Yin RL, Jiang WQ, Wang JJ, Wang ZY (2014). Selection and validation of suitable reference genes in skin tissue of Liaoning cashmere goat during hair follicle cycle. Livest Sci.

[11] Gao Y, Wang X, Yan H, Zeng J, Ma S, Niu Y (2016). Comparative Transcriptome analysis of fetal skin reveals key genes related to hair follicle morphogenesis in cashmere goats. PLoS One.

[12] Wang X, Liu J, Zhou G, Guo J, Yan H, Niu Y (2016). Whole-genome sequencing of eight goat populations for the detection of selection signatures underlying production and adaptive traits. Sci Rep.

[13] Li X, Su R, Wan W, Zhang W, Jiang H, Qiao X (2017). Identification of selection signals by large-scale whole-genome resequencing of cashmere goats. Sci Rep.

[14] Geng R, Wang L, Wang X, Chen Y (2014). Cyclic expression of Lhx2 is involved in secondary hair follicle development in cashmere goat. Gene Expr Patterns.

[15] Wang X, Cai B, Zhou J, Zhu H, Niu Y, Ma B (2016). Disruption of FGF5 in cashmere goats using CRISPR/Cas9 results in more secondary hair follicles and longer fibers. PLoS One.

[16] Bai WL, Wang JJ, Yin RH, Dang YL, Wang ZY, Zhu YB (2017). Molecular characterization of HOXC8 gene and methylation status analysis of its exon 1 associated with the length of cashmere fiber in Liaoning cashmere goat. Genetica..

[17] Su R, Fan Y, Qiao X, Li X, Zhang L, Li C (2018). Transcriptomic analysis reveals critical genes for the hair follicle of Inner Mongolia cashmere goat from catagen to telogen. PLoS One.

[18] Palombo V, Milanesi M, Sgorlon S, Capomaccio S, Mele M, Nicolazzi E (2018). Genome-wide association study of milk fatty acid composition in Italian Simmental and Italian Holstein cows using single nucleotide polymorphism arrays. J Dairy Sci.

[19] Foitzik K, Krause K, Nixon AJ, Ford CA, Ohnemus U, Pearson AJ (2003). Prolactin and its receptor are expressed in murine hair follicle epithelium, show hair cycle-dependent expression, and induce Catagen. Am J Pathol.

[20] Celi P, Seren E, Celi R, Parmeggiani A, Trana AD (2003). Relationships between blood hormonal concentrations and secondary fibre shedding in young cashmere-bearing goats at their first moult. Anim Sci.

[21] Wu J, Li Y, Gong H, Wu D, Li C, Liu B. Circannual Rhythm in the Skin Gene Expression of Cashmere Goat bioRxiv. 2020;:2020.04.04.023044.

[22] Severin RK, Li X, Qian K, Mueller AC, Petukhova L. Computational derivation of a molecular framework for hair follicle biology from disease genes. Sci Rep. 2017;7. 10.1038/s41598-017-16050-9. 10.1038/s41598-017-16050-9 PMC5701154 29176608

[23] Yang L, Peng R (2010). Unveiling hair follicle stem cells. Stem Cell Rev Rep.

[24] Vahidnezhad H, Youssefian L, Uitto J (2016). Molecular genetics of the PI3K-AKT-mTOR pathway in Genodermatoses: diagnostic implications and treatment opportunities. J Investig Dermatol.

[25] Ma KL, Nyamtsengel V, Bao WL, Lian MY, Wang WP, Wang YF (2014). Overexpression of protein kinase B/AKT induces phosphorylation of p70S6K and 4E-BP1 in goat fetal fibroblasts. Genet Mol Res.

[26] Schaaij-Visser TBM, Bremmer JF, Braakhuis BJM, Heck AJR, Slijper M, van der Waal I (2010). Evaluation of cornulin, keratin 4, keratin 13 expression and grade of dysplasia for predicting malignant progression of oral leukoplakia. Oral Oncol.

[27] Mietton L, Lebrun N, Giurgea I, Goldenberg A, Saintpierre B, Hamroune J (2018). RNA sequencing and pathway analysis identify important pathways involved in Hypertrichosis and intellectual disability in patients with Wiedemann–Steiner syndrome. NeuroMolecular Med.

[28] Paramio J. Intermediate filaments. Landes Bioscience and Springer Science+ Business Media. LLC; 2006.

[29] Zhu B, Guo Z, Jin M, Bai Y, Yang W, Hui L (2018). Establishment of dermal sheath cell line from cashmere goat and characterizing cytokeratin 13 as its novel biomarker. Biotechnol Lett.

[30] Prunotto M, Farina A, Lane L, Pernin A, Schifferli J, Hochstrasser DF (2013). Proteomic analysis of podocyte exosome-enriched fraction from normal human urine. J Proteome.

[31] Jaworski CJ, Aryankalayil-John M, Campos MM, Fariss RN, Rowsey J, Agarwalla N (2009). Expression analysis of human pterygium shows a predominance of conjunctival and limbal markers and genes associated with cell migration. Mol Vis.

[32] Marchini G, Ståbi B, Kankes K, Lonne-Rahm S, Østergaard M, Nielsen S (2003). AQP1 and AQP3, Psoriasin, and nitric oxide synthases 1–3 are inflammatory mediators in erythema Toxicum Neonatorum. Pediatr Dermatol.

[33] Almohanna HM, Ahmed AA, Tsatalis JP, Tosti A (2019). The role of vitamins and minerals in hair loss: a review. Dermatol Ther (Heidelb).

[34] Jafri SK, Kumar R, Lashari SK, Chand P. Menkes disease: A rare disorder. J Pak Med Assoc. 2017;67(10):1609–11. 28955085

[35] Pazzaglia I, Mercati F, Antonini M, Capomaccio S, Cappelli K, Dall’Aglio C (2019). PDGFA in cashmere goat: a motivation for the hair follicle stem cells to activate. Animals..

[36] Inoue K, Yoshimura K, Helgason CD, Miller CL (2013). Isolation and characterization of human hair follicle epithelial cells. Basic Cell Culture Protocols.

[37] Ohyama M, Kobayashi T, Singh SR (2012). Isolation and characterization of stem cell-enriched human and canine hair follicle keratinocytes. Somatic stem cells: methods and protocols.

[38] Mercati F, Dall’Aglio C, Timperi L, Scocco P, Felice ED, Maranesi M. Epithelial expression of the hormone leptin by bovine skin. Eur J Histochem. 2019;63. 10.4081/ejh.2019.2993. 10.4081/ejh.2019.2993 PMC6340309 30652436

[39] Ryder ML, Stephenson SK. Wool growth.,(Academic Press: London). New York. 1968.

[40] Bolger AM, Lohse M, Usadel B (2014). Trimmomatic: a flexible trimmer for Illumina sequence data. Bioinformatics..

[41] Dobin A, Davis CA, Schlesinger F, Drenkow J, Zaleski C, Jha S (2013). STAR: ultrafast universal RNA-seq aligner. Bioinformatics..

[42] Maere S, Heymans K, Kuiper M (2005). BiNGO: a Cytoscape plugin to assess overrepresentation of gene ontology categories in biological networks. Bioinformatics..

[43] Kanehisa M, Goto S (2000). KEGG: Kyoto encyclopedia of genes and genomes. Nucleic Acids Res.

[44] Markham NR, Zuker M, Keith JM (2008). UNAFold. Bioinformatics: structure, function and applications.

