# Supplementary material for: Shedding light on cashmere goat hair follicle biology: from morphology analyses to transcriptomic landascape
Source: BMC Genomics. 2020 Jul 2;21:458. doi: 10.1186/s12864-020-06870-x (PMC7330943; doi:10.1186/s12864-020-06870-x)
Supplement: Supplementary file 1 — Additional file 1. Output of trimming and mapping. [file 12864_2020_6870_MOESM1_ESM.docx]

**Additional file 1: Output of trimming and mapping**

Table 1 Input and output of trimming procedure for each sample

| **TRIMMING** | | | |
| --- | --- | --- | --- |
| **SAMPLE** | **RAW READ PAIRS** | **OUTPUT** | **% SURVIVING** |
| **ANA_01** | 44760537 | 39313683 | 87.83% |
| **ANA_02** | 45256694,00 | 39124102 | 86.45% |
| **ANA_03** | 37241153,00 | 31835423 | 85.48% |
| **ANA_04** | 34325994,00 | 24028982 | 70.00% |
| **ANA_05** | 36276269,00 | 33635995 | 92.72% |
| **CATA_01** | 51838184,00 | 50258628 | 96.95% |
| **CATA_02** | 54113894,00 | 52156413 | 96.38% |
| **CATA_03** | 50313077,00 | 49263209 | 97.91% |
| **CATA_04** | 39805722,00 | 39330501 | 98.81% |
| **CATA_05** | 39444140,00 | 38340413 | 97.20% |

Table 2 Cleaned reads mapped inARS1 genome for each sample

| **MAPPING** | | |
| --- | --- | --- |
| **SAMPLE** | **Uniquely mapped reads number** | **% GENOME MAPPED** |
| **ANA_01** | 32438012 | 82.51% |
| **ANA_02** | 31203503 | 79.76% |
| **ANA_03** | 26629555 | 83.65% |
| **ANA_04** | 20255381 | 84.30% |
| **ANA_05** | 28973835 | 86.14% |
| **CATA_01** | 44623668 | 88.79% |
| **CATA_02** | 44868077 | 86.03% |
| **CATA_03** | 43077502 | 87.44% |
| **CATA_04** | 34265835 | 87.12% |
| **CATA_05** | 34382053 | 89.68% |
