# Supplementary material for: Shedding light on cashmere goat hair follicle biology: from morphology analyses to transcriptomic landascape
Source: BMC Genomics. 2020 Jul 2;21:458. doi: 10.1186/s12864-020-06870-x (PMC7330943; doi:10.1186/s12864-020-06870-x)
Supplement: Supplementary file 2 — Additional file 2. List of DEGs. [file 12864_2020_6870_MOESM2_ESM.docx]

**Additional file 2: List of DEGs**

| **GeneID** | **Gene Name** | **logFC** | **logCPM** | **P-Value** | **FDR** |
| --- | --- | --- | --- | --- | --- |
| ENSCHIG00000021789 | S100A7A | -6,05 | 7,70 | 2,21E-12 | 1,95E-10 |
| ENSCHIG00000013424 | LOC102191570 | -5,74 | 3,57 | 6,91E-21 | 3,92E-18 |
| ENSCHIG00000026589 | KRT4 | -5,70 | 7,31 | 3,23E-40 | 1,65E-36 |
| ENSCHIG00000026269 | TYRP1 | -4,28 | 3,66 | 3,92E-07 | 7,60E-06 |
| ENSCHIG00000013983 | SPINK7 | -3,89 | 7,16 | 4,14E-49 | 5,17E-45 |
| ENSCHIG00000021319 | COL1A1 | -3,56 | 7,68 | 2,83E-04 | 1,83E-03 |
| ENSCHIG00000021338 | LOC102169411 | -3,56 | 6,11 | 1,18E-07 | 2,72E-06 |
| ENSCHIG00000024875 | PMEL | -3,31 | 4,73 | 1,83E-11 | 1,31E-09 |
| ENSCHIG00000025217 | RGS4 | -3,26 | 2,12 | 7,66E-10 | 3,69E-08 |
| ENSCHIG00000025647 | COL1A2 | -3,01 | 7,56 | 1,00E-03 | 5,09E-03 |
| ENSCHIG00000012522 | IL20 | -2,89 | 2,91 | 1,45E-10 | 8,29E-09 |
| ENSCHIG00000021187 | AQP3 | -2,87 | 9,34 | 3,96E-40 | 1,65E-36 |
| ENSCHIG00000027066 | SOCS2 | -2,80 | 3,87 | 3,06E-29 | 6,38E-26 |
| ENSCHIG00000019803 | KRT13 | -2,80 | 4,65 | 3,53E-08 | 9,91E-07 |
| ENSCHIG00000014595 | LYPD2 | -2,79 | 2,71 | 2,19E-18 | 7,20E-16 |
| ENSCHIG00000027083 | LOC102176161 | -2,72 | 5,25 | 1,29E-11 | 9,54E-10 |
| ENSCHIG00000014493 | MLANA | -2,66 | 2,37 | 1,12E-10 | 6,63E-09 |
| ENSCHIG00000011929 | TNFSF18 | -2,65 | 2,42 | 1,77E-14 | 2,66E-12 |
| ENSCHIG00000004685 | TGFBI | -2,58 | 5,74 | 7,85E-17 | 1,81E-14 |
| ENSCHIG00000009412 | AQP5 | -2,51 | 1,68 | 6,81E-08 | 1,69E-06 |
| ENSCHIG00000025261 | ALDH3A1 | -2,42 | 1,93 | 1,43E-08 | 4,51E-07 |
| ENSCHIG00000015017 | LOC108636550 | -2,38 | 3,00 | 6,73E-08 | 1,69E-06 |
| ENSCHIG00000017958 | ALDH1A1 | -2,38 | 5,84 | 4,98E-19 | 1,94E-16 |
| ENSCHIG00000009143 | ACTG2 | -2,33 | 2,69 | 9,64E-05 | 7,48E-04 |
| ENSCHIG00000012291 | IL6 | -2,25 | 3,08 | 7,63E-05 | 6,24E-04 |
| ENSCHIG00000017221 | TMEM266 | -2,24 | 1,82 | 6,02E-09 | 2,17E-07 |
| ENSCHIG00000010779 | LOC108633460 | -2,21 | 3,82 | 1,58E-11 | 1,14E-09 |
| ENSCHIG00000010817 | HIST1H1D | -2,19 | 4,26 | 3,98E-28 | 7,10E-25 |
| ENSCHIG00000001557 | CCDC80 | -2,19 | 4,21 | 4,12E-03 | 1,59E-02 |
| ENSCHIG00000008973 | TRPM1 | -2,15 | 3,29 | 1,97E-09 | 8,35E-08 |
| ENSCHIG00000010389 | SPINK4 | -2,15 | 3,81 | 4,55E-09 | 1,70E-07 |
| ENSCHIG00000012357 | KRT38 | -2,13 | 4,34 | 1,16E-06 | 1,90E-05 |
| ENSCHIG00000006002 | LOC106502686 | -2,11 | 1,40 | 4,86E-05 | 4,35E-04 |
| ENSCHIG00000014961 | ADAMTS14 | -2,11 | 2,31 | 1,57E-08 | 4,83E-07 |
| ENSCHIG00000025767 | CSRP2 | -2,11 | 4,59 | 1,28E-23 | 1,14E-20 |
| ENSCHIG00000010344 | LOC108636554 | -2,09 | 1,81 | 1,81E-04 | 1,25E-03 |
| ENSCHIG00000013432 | PDK4 | -2,09 | 8,76 | 2,10E-11 | 1,44E-09 |
| ENSCHIG00000017063 | IFIT2 | -2,08 | 1,65 | 4,32E-11 | 2,77E-09 |
| ENSCHIG00000014257 | STMN2 | -2,07 | 2,04 | 9,02E-04 | 4,68E-03 |
| ENSCHIG00000024614 | PTGES | -2,07 | 2,67 | 3,82E-15 | 6,19E-13 |
| ENSCHIG00000000726 | TMEM106A | -2,04 | 1,48 | 9,77E-12 | 7,48E-10 |
| ENSCHIG00000012829 | PSMB8 | -2,03 | 4,78 | 1,29E-27 | 1,79E-24 |
| ENSCHIG00000011284 | LOC102181348 | -2,02 | 2,35 | 6,97E-10 | 3,38E-08 |
| ENSCHIG00000011548 | LOC108636548 | -2,02 | 1,95 | 2,72E-05 | 2,66E-04 |
| ENSCHIG00000021248 | TPPP3 | -1,97 | 6,44 | 5,00E-34 | 1,56E-30 |
| ENSCHIG00000021513 | PSMB9 | -1,97 | 3,64 | 8,29E-23 | 6,09E-20 |
| ENSCHIG00000009614 | LOC108633459 | -1,96 | 6,39 | 2,09E-05 | 2,14E-04 |
| ENSCHIG00000024469 | IL1B | -1,96 | 2,26 | 4,58E-04 | 2,72E-03 |
| ENSCHIG00000017373 | LOC108636552 | -1,95 | 2,08 | 8,13E-05 | 6,53E-04 |
| ENSCHIG00000018312 | COL6A3 | -1,94 | 2,91 | 1,67E-03 | 7,73E-03 |
| ENSCHIG00000004030 | CH25H | -1,94 | 2,03 | 9,40E-10 | 4,38E-08 |
| ENSCHIG00000000369 | YDJC | -1,93 | 2,27 | 1,71E-12 | 1,55E-10 |
| ENSCHIG00000011267 | LRRC36 | -1,91 | 1,16 | 2,78E-08 | 8,04E-07 |
| ENSCHIG00000024310 | IGFBP3 | -1,90 | 5,52 | 3,08E-20 | 1,48E-17 |
| ENSCHIG00000004889 | COL6A1 | -1,88 | 3,76 | 3,83E-03 | 1,50E-02 |
| ENSCHIG00000011241 | LOC102174264 | -1,86 | 3,68 | 2,60E-03 | 1,10E-02 |
| ENSCHIG00000000407 | CRIP1 | -1,85 | 3,44 | 1,45E-06 | 2,29E-05 |
| ENSCHIG00000007511 | HIC2 | -1,84 | 2,54 | 1,57E-07 | 3,48E-06 |
| ENSCHIG00000025438 | PLP1 | -1,83 | 2,59 | 4,53E-05 | 4,10E-04 |
| ENSCHIG00000013691 | PCOLCE | -1,81 | 2,74 | 5,24E-03 | 1,93E-02 |
| ENSCHIG00000016589 | EEF1A2 | -1,80 | 1,44 | 7,07E-06 | 8,79E-05 |
| ENSCHIG00000026621 | S100A8 | -1,80 | 3,62 | 1,01E-03 | 5,12E-03 |
| ENSCHIG00000023197 | PODXL2 | -1,78 | 2,40 | 6,40E-07 | 1,16E-05 |
| ENSCHIG00000017858 | IFI6 | -1,77 | 2,70 | 1,15E-07 | 2,67E-06 |
| ENSCHIG00000014081 | LSP1 | -1,77 | 2,55 | 7,36E-07 | 1,30E-05 |
| ENSCHIG00000026012 | LOC102188567 | -1,75 | 5,65 | 2,03E-05 | 2,09E-04 |
| ENSCHIG00000018153 | LOC108633505 | -1,74 | 7,15 | 7,68E-08 | 1,87E-06 |
| ENSCHIG00000017708 | TSPEAR | -1,73 | 1,38 | 2,37E-04 | 1,56E-03 |
| ENSCHIG00000004792 | HIST1H2AC | -1,73 | 1,76 | 1,11E-05 | 1,25E-04 |
| ENSCHIG00000021004 | ITIH5 | -1,73 | 5,65 | 6,43E-12 | 5,18E-10 |
| ENSCHIG00000006854 | CLEC3B | -1,72 | 4,02 | 2,12E-03 | 9,38E-03 |
| ENSCHIG00000016288 | SERPINE2 | -1,72 | 3,82 | 3,64E-07 | 7,09E-06 |
| ENSCHIG00000014084 | RUNX1T1 | -1,72 | 1,10 | 1,39E-06 | 2,21E-05 |
| ENSCHIG00000023567 | CCL2 | -1,72 | 1,34 | 6,82E-05 | 5,70E-04 |
| ENSCHIG00000023487 | EPSTI1 | -1,72 | 2,23 | 2,69E-09 | 1,10E-07 |
| ENSCHIG00000019467 | LOC102181202 | -1,71 | 8,96 | 3,76E-14 | 5,28E-12 |
| ENSCHIG00000024813 | LOC102172766 | -1,69 | 4,38 | 1,63E-05 | 1,73E-04 |
| ENSCHIG00000023756 | PLIN3 | -1,68 | 5,45 | 1,83E-15 | 3,26E-13 |
| ENSCHIG00000016060 | LOC106501751 | -1,68 | 3,31 | 7,95E-16 | 1,58E-13 |
| ENSCHIG00000022150 | DBP | -1,68 | 5,80 | 2,67E-22 | 1,85E-19 |
| ENSCHIG00000014590 | TPM2 | -1,65 | 2,13 | 7,66E-05 | 6,25E-04 |
| ENSCHIG00000014318 | ARPP21 | -1,63 | 3,06 | 1,69E-05 | 1,79E-04 |
| ENSCHIG00000008896 | PTP4A3 | -1,63 | 1,14 | 1,52E-05 | 1,64E-04 |
| ENSCHIG00000007736 | IFITM3 | -1,62 | 3,29 | 3,83E-10 | 2,02E-08 |
| ENSCHIG00000009921 | ZNF575 | -1,61 | 1,32 | 9,81E-05 | 7,58E-04 |
| ENSCHIG00000020950 | METTL27 | -1,59 | 3,94 | 2,66E-10 | 1,44E-08 |
| ENSCHIG00000006387 | HIST1H1E | -1,58 | 1,24 | 2,27E-06 | 3,36E-05 |
| ENSCHIG00000021938 | LOC102182692 | -1,58 | 6,24 | 1,37E-26 | 1,55E-23 |
| ENSCHIG00000019116 | LOC102189655 | -1,57 | 3,34 | 2,61E-04 | 1,71E-03 |
| ENSCHIG00000021115 | AHSG | -1,56 | 0,66 | 2,13E-04 | 1,43E-03 |
| ENSCHIG00000016652 | CRISPLD1 | -1,55 | 3,63 | 4,40E-07 | 8,38E-06 |
| ENSCHIG00000011883 | EDN1 | -1,55 | 4,36 | 2,63E-13 | 2,96E-11 |
| ENSCHIG00000002653 | TNFRSF18 | -1,54 | 5,24 | 2,88E-11 | 1,93E-09 |
| ENSCHIG00000012437 | SLC6A1 | -1,53 | 1,88 | 2,03E-06 | 3,07E-05 |
| ENSCHIG00000024785 | SLC6A6 | -1,52 | 3,70 | 9,72E-12 | 7,48E-10 |
| ENSCHIG00000023014 | DENND2A | -1,52 | 1,07 | 6,03E-04 | 3,37E-03 |
| ENSCHIG00000016336 | RNF144A | -1,51 | 1,67 | 1,37E-03 | 6,57E-03 |
| ENSCHIG00000025976 | DUSP2 | 1,50 | 3,02 | 1,98E-06 | 2,99E-05 |
| ENSCHIG00000004376 | CDK5R1 | 1,50 | 1,22 | 2,13E-06 | 3,19E-05 |
| ENSCHIG00000009374 | MIR4657 | 1,50 | 1,94 | 2,29E-08 | 6,76E-07 |
| ENSCHIG00000019978 | SMPD3 | 1,50 | 3,49 | 5,82E-06 | 7,43E-05 |
| ENSCHIG00000014907 | DHCR7 | 1,50 | 5,06 | 3,33E-17 | 8,15E-15 |
| ENSCHIG00000020591 | TMEM86A | 1,51 | 4,82 | 1,12E-15 | 2,09E-13 |
| ENSCHIG00000014366 | SETBP1 | 1,51 | 3,89 | 1,73E-09 | 7,52E-08 |
| ENSCHIG00000026791 | GSDME | 1,54 | 2,66 | 5,64E-05 | 4,89E-04 |
| ENSCHIG00000024023 | MARCH3 | 1,54 | 4,17 | 1,06E-16 | 2,40E-14 |
| ENSCHIG00000012088 | LOC108633223 | 1,55 | 7,66 | 5,57E-04 | 3,15E-03 |
| ENSCHIG00000024864 | DLX2 | 1,55 | 2,61 | 5,19E-11 | 3,29E-09 |
| ENSCHIG00000000814 | SLC45A3 | 1,55 | 4,05 | 7,99E-08 | 1,95E-06 |
| ENSCHIG00000005294 | CD5 | 1,56 | 1,39 | 1,88E-03 | 8,50E-03 |
| ENSCHIG00000023010 | PITPNC1 | 1,56 | 2,17 | 4,71E-09 | 1,75E-07 |
| ENSCHIG00000008722 | RF00272 | 1,56 | 2,77 | 2,91E-07 | 5,90E-06 |
| ENSCHIG00000016448 | ACE2 | 1,57 | 4,08 | 8,11E-09 | 2,81E-07 |
| ENSCHIG00000016134 | SERPINA12 | 1,57 | 6,73 | 1,63E-08 | 5,00E-07 |
| ENSCHIG00000010320 | DGAT2 | 1,59 | 7,99 | 2,96E-10 | 1,59E-08 |
| ENSCHIG00000000448 | BMPR1A | 1,59 | 5,46 | 7,57E-28 | 1,18E-24 |
| ENSCHIG00000004800 | PSAPL1 | 1,60 | 6,19 | 3,89E-08 | 1,08E-06 |
| ENSCHIG00000019461 | SMAD7 | 1,60 | 5,29 | 4,37E-19 | 1,76E-16 |
| ENSCHIG00000026756 | COL27A1 | 1,60 | 2,18 | 3,87E-05 | 3,61E-04 |
| ENSCHIG00000016601 | SLC16A1 | 1,60 | 2,51 | 4,02E-08 | 1,10E-06 |
| ENSCHIG00000022637 | PAK3 | 1,60 | 2,21 | 3,70E-08 | 1,03E-06 |
| ENSCHIG00000015394 | PPP4R4 | 1,61 | 2,94 | 1,15E-08 | 3,73E-07 |
| ENSCHIG00000026965 | HEY2 | 1,62 | 2,26 | 1,11E-08 | 3,64E-07 |
| ENSCHIG00000026694 | NRCAM | 1,62 | 2,59 | 3,11E-07 | 6,22E-06 |
| ENSCHIG00000026015 | KRT78 | 1,62 | 3,90 | 5,09E-07 | 9,55E-06 |
| ENSCHIG00000013627 | ASPRV1 | 1,63 | 7,59 | 1,65E-15 | 3,02E-13 |
| ENSCHIG00000016540 | SLC38A4 | 1,63 | 2,90 | 4,11E-10 | 2,15E-08 |
| ENSCHIG00000011375 | CHRD | 1,64 | 1,78 | 1,48E-05 | 1,60E-04 |
| ENSCHIG00000020570 | LOC102188176 | 1,64 | 3,87 | 1,55E-05 | 1,67E-04 |
| ENSCHIG00000026319 | LOC108638285 | 1,65 | 5,44 | 9,03E-04 | 4,69E-03 |
| ENSCHIG00000012961 | PPM1H | 1,65 | 1,89 | 1,21E-07 | 2,76E-06 |
| ENSCHIG00000026727 | SDCBP2 | 1,67 | 2,34 | 8,15E-08 | 1,98E-06 |
| ENSCHIG00000003190 | XK | 1,67 | 1,81 | 1,00E-05 | 1,16E-04 |
| ENSCHIG00000016209 | SLC19A3 | 1,68 | 1,79 | 1,08E-04 | 8,18E-04 |
| ENSCHIG00000026715 | GUSB | 1,68 | 5,78 | 9,79E-09 | 3,28E-07 |
| ENSCHIG00000003599 | RF00554 | 1,68 | 2,55 | 1,96E-10 | 1,09E-08 |
| ENSCHIG00000026902 | GGCT | 1,69 | 5,86 | 2,20E-13 | 2,54E-11 |
| ENSCHIG00000001690 | CA6 | 1,69 | 2,71 | 1,03E-05 | 1,18E-04 |
| ENSCHIG00000020127 | SCN9A | 1,69 | 3,59 | 6,13E-07 | 1,11E-05 |
| ENSCHIG00000018377 | SEMA3D | 1,69 | 3,11 | 4,78E-10 | 2,46E-08 |
| ENSCHIG00000019849 | SLC7A5 | 1,74 | 5,26 | 2,37E-19 | 9,85E-17 |
| ENSCHIG00000013100 | KRT23 | 1,76 | 6,89 | 1,05E-11 | 7,98E-10 |
| ENSCHIG00000007676 | PLS1 | 1,78 | 1,63 | 6,29E-04 | 3,48E-03 |
| ENSCHIG00000011933 | PLEKHG1 | 1,78 | 5,34 | 8,90E-27 | 1,11E-23 |
| ENSCHIG00000012134 | FA2H | 1,78 | 7,12 | 5,36E-06 | 6,90E-05 |
| ENSCHIG00000003120 | RF00284 | 1,79 | 2,18 | 2,02E-11 | 1,40E-09 |
| ENSCHIG00000013304 | GABRP | 1,79 | 5,40 | 5,67E-18 | 1,71E-15 |
| ENSCHIG00000017259 | ELOVL4 | 1,80 | 7,33 | 5,15E-16 | 1,05E-13 |
| ENSCHIG00000026468 | MMP7 | 1,81 | 3,03 | 6,78E-08 | 1,69E-06 |
| ENSCHIG00000023966 | ABHD12B | 1,81 | 1,86 | 6,53E-08 | 1,64E-06 |
| ENSCHIG00000023208 | FAR2 | 1,82 | 7,30 | 5,86E-08 | 1,51E-06 |
| ENSCHIG00000021123 | LOC102187504 | 1,82 | 1,67 | 9,91E-04 | 5,06E-03 |
| ENSCHIG00000018867 | PTPRU | 1,82 | 2,13 | 3,01E-06 | 4,25E-05 |
| ENSCHIG00000024519 | ACSM3 | 1,83 | 4,68 | 1,89E-09 | 8,10E-08 |
| ENSCHIG00000013488 | CALN1 | 1,83 | 1,70 | 1,59E-08 | 4,89E-07 |
| ENSCHIG00000012019 | LOC102176090 | 1,83 | 1,28 | 1,03E-06 | 1,73E-05 |
| ENSCHIG00000026018 | FREM1 | 1,85 | 1,17 | 1,14E-04 | 8,57E-04 |
| ENSCHIG00000012020 | EMB | 1,85 | 2,26 | 2,84E-07 | 5,77E-06 |
| ENSCHIG00000010391 | THRSP | 1,85 | 3,45 | 2,08E-05 | 2,13E-04 |
| ENSCHIG00000015313 | LOC102190037 | 1,86 | 8,26 | 3,95E-06 | 5,32E-05 |
| ENSCHIG00000000727 | RF00221 | 1,90 | 1,53 | 1,02E-05 | 1,17E-04 |
| ENSCHIG00000003901 | GJA1 | 1,90 | 8,35 | 7,42E-32 | 1,85E-28 |
| ENSCHIG00000007829 | PDP2 | 1,91 | 3,14 | 1,62E-13 | 1,97E-11 |
| ENSCHIG00000024549 | ADCY1 | 1,91 | 2,94 | 4,92E-08 | 1,31E-06 |
| ENSCHIG00000008294 | NSG1 | 1,91 | 1,89 | 2,11E-10 | 1,16E-08 |
| ENSCHIG00000013747 | LYPD5 | 1,91 | 6,82 | 1,88E-11 | 1,33E-09 |
| ENSCHIG00000015186 | GFRA1 | 1,92 | 1,33 | 7,45E-07 | 1,32E-05 |
| ENSCHIG00000019609 | MC5R | 1,95 | 2,30 | 1,23E-08 | 3,95E-07 |
| ENSCHIG00000008392 | CACNA1G | 1,95 | 1,09 | 1,91E-08 | 5,72E-07 |
| ENSCHIG00000010101 | MYZAP | 1,96 | 4,13 | 1,88E-15 | 3,31E-13 |
| ENSCHIG00000020567 | ELOVL3 | 1,97 | 7,50 | 5,68E-10 | 2,85E-08 |
| ENSCHIG00000018895 | LOC102183358 | 1,99 | 1,61 | 8,95E-06 | 1,06E-04 |
| ENSCHIG00000003245 | WFDC5 | 2,00 | 2,06 | 4,24E-08 | 1,15E-06 |
| ENSCHIG00000011643 | RGN | 2,00 | 4,72 | 5,18E-19 | 1,96E-16 |
| ENSCHIG00000016974 | SCN3A | 2,00 | 2,31 | 2,26E-03 | 9,87E-03 |
| ENSCHIG00000021152 | SLC6A14 | 2,03 | 5,41 | 7,05E-22 | 4,64E-19 |
| ENSCHIG00000018974 | LRP2 | 2,04 | 3,91 | 7,90E-07 | 1,38E-05 |
| ENSCHIG00000015258 | IGF2BP2 | 2,04 | 2,11 | 5,32E-07 | 9,91E-06 |
| ENSCHIG00000019917 | TRPM6 | 2,06 | 1,37 | 1,76E-07 | 3,84E-06 |
| ENSCHIG00000008644 | CDSN | 2,11 | 8,42 | 2,40E-20 | 1,20E-17 |
| ENSCHIG00000021773 | CEMIP | 2,11 | 1,93 | 8,83E-12 | 6,89E-10 |
| ENSCHIG00000021710 | IL36RN | 2,13 | 4,98 | 1,56E-18 | 5,68E-16 |
| ENSCHIG00000014126 | SLC10A6 | 2,13 | 1,86 | 1,94E-10 | 1,09E-08 |
| ENSCHIG00000026887 | CLDN8 | 2,15 | 4,20 | 1,09E-15 | 2,06E-13 |
| ENSCHIG00000013351 | SLC37A2 | 2,15 | 2,61 | 1,71E-09 | 7,45E-08 |
| ENSCHIG00000012426 | LOC102181382 | 2,15 | 1,92 | 1,09E-08 | 3,60E-07 |
| ENSCHIG00000011672 | NIPAL4 | 2,15 | 4,93 | 7,51E-18 | 2,13E-15 |
| ENSCHIG00000023870 | KPRP | 2,16 | 4,70 | 7,90E-07 | 1,38E-05 |
| ENSCHIG00000015181 | TMPRSS11F | 2,17 | 3,77 | 1,66E-12 | 1,52E-10 |
| ENSCHIG00000016088 | LOC102187755 | 2,19 | 6,45 | 1,25E-05 | 1,40E-04 |
| ENSCHIG00000014043 | YOD1 | 2,23 | 5,04 | 1,97E-18 | 6,82E-16 |
| ENSCHIG00000024394 | ATP6V0A4 | 2,23 | 3,89 | 1,37E-17 | 3,63E-15 |
| ENSCHIG00000021557 | SLC46A2 | 2,35 | 3,97 | 1,72E-13 | 2,06E-11 |
| ENSCHIG00000020018 | SPINK6 | 2,37 | 3,24 | 3,30E-05 | 3,16E-04 |
| ENSCHIG00000013721 | SLC36A2 | 2,38 | 3,83 | 5,74E-18 | 1,71E-15 |
| ENSCHIG00000021985 | LOC102187932 | 2,42 | 4,25 | 6,70E-06 | 8,37E-05 |
| ENSCHIG00000023029 | TUBA8 | 2,42 | 3,55 | 3,41E-10 | 1,81E-08 |
| ENSCHIG00000026251 | MBOAT2 | 2,44 | 5,83 | 1,08E-16 | 2,40E-14 |
| ENSCHIG00000021424 | SOAT1 | 2,54 | 6,40 | 4,53E-12 | 3,74E-10 |
| ENSCHIG00000020836 | LGALS15 | 2,57 | 1,38 | 1,04E-09 | 4,76E-08 |
| ENSCHIG00000019630 | VTCN1 | 2,63 | 2,12 | 8,60E-11 | 5,16E-09 |
| ENSCHIG00000024516 | LOC102184404 | 2,68 | 9,08 | 2,08E-11 | 1,43E-09 |
| ENSCHIG00000004964 | TPRG1 | 2,79 | 5,05 | 1,57E-20 | 8,19E-18 |
| ENSCHIG00000010930 | ELF3 | 2,81 | 3,03 | 3,18E-11 | 2,08E-09 |
| ENSCHIG00000006757 | SERPINB7 | 2,82 | 5,17 | 2,42E-23 | 2,01E-20 |
| ENSCHIG00000010326 | PLIN4 | 2,83 | 2,67 | 1,62E-10 | 9,15E-09 |
| ENSCHIG00000025514 | CPM | 2,91 | 4,42 | 1,91E-25 | 1,99E-22 |
| ENSCHIG00000003716 | IL37 | 2,92 | 4,97 | 2,20E-17 | 5,50E-15 |
| ENSCHIG00000017048 | SLC9A2 | 3,16 | 1,98 | 9,39E-21 | 5,10E-18 |
| ENSCHIG00000022064 | GLDC | 3,19 | 2,04 | 2,39E-12 | 2,08E-10 |
| ENSCHIG00000023059 | KIAA1324L | 3,36 | 2,24 | 3,65E-17 | 8,76E-15 |
| ENSCHIG00000017002 | CYP4X1 | 3,65 | 6,49 | 2,46E-13 | 2,82E-11 |
| ENSCHIG00000024689 | TNFRSF11B | 4,37 | 3,21 | 2,62E-13 | 2,96E-11 |
| ENSCHIG00000026820 | CERULOPLASMIN | 7,28 | 2,26 | 1,08E-19 | 4,82E-17 |
