# Supplementary material for: Shedding light on cashmere goat hair follicle biology: from morphology analyses to transcriptomic landascape
Source: BMC Genomics. 2020 Jul 2;21:458. doi: 10.1186/s12864-020-06870-x (PMC7330943; doi:10.1186/s12864-020-06870-x)
Supplement: Supplementary file 3 — Additional file 3. Output of significant gene enrichment processed by PIA. [file 12864_2020_6870_MOESM3_ESM.docx]

**Additional file 3: Output of significant gene enrichment processed by PIA**

| **N GENES** | **ALL GENES** | **P VALUE** | **PADJ** | **PATHWAY NAME** |
| --- | --- | --- | --- | --- |
| 979 | 1370 | 2,28E-29 | 7,45E-26 | Metabolic pathways |
| 156 | 168 | 6,31E-14 | 1,03E-11 | Protein processing in endoplasmic reticulum |
| 199 | 233 | 1,07E-09 | 1,16E-07 | Thermogenesis |
| 118 | 125 | 4,13E-10 | 3,36E-07 | Cell cycle |
| 146 | 163 | 4,07E-08 | 2,66E-06 | Cellular senescence |
| 211 | 259 | 8,57E-06 | 4,65E-04 | Endocytosis |
| 149 | 175 | 9,35E-05 | 4,35E-02 | Alzheimer disease |
| 120 | 137 | 1,39E-02 | 5,67E-01 | Spliceosome |
| 125 | 145 | 5,39E-02 | 1,95E+00 | Ubiquitin mediated proteolysis |
| 142 | 170 | 1,72E-01 | 5,62E+00 | RNA transport |
| 115 | 133 | 5,08E-01 | 1,51E-01 | Autophagy - animal |
| 377 | 533 | 1,13E+00 | 3,06E+01 | Pathways in cancer |
| 157 | 194 | 1,58E+00 | 3,95E+01 | Huntington disease |
| 117 | 138 | 6,32E+00 | 1,47E+01 | Oxidative phosphorylation |
| 104 | 120 | 7,56E+00 | 1,64E+02 | Neurotrophin signaling pathway |
| 205 | 268 | 1,00E+01 | 2,05E+02 | Human T-cell leukemia virus 1 infection |
| 119 | 143 | 5,97E+01 | 1,15E+03 | Fluid shear stress and atherosclerosis |
| 70 | 76 | 1,12E+02 | 2,03E+03 | Chronic myeloid leukemia |
| 119 | 144 | 1,58E+02 | 2,72E+03 | Parkinson disease |
| 81 | 91 | 1,90E+01 | 3,10E+03 | Colorectal cancer |
| 222 | 299 | 2,15E+02 | 3,33E+03 | MAPK signaling pathway |
| 106 | 127 | 7,72E+02 | 1,13E+04 | Lysosome |
| 124 | 153 | 7,96E+02 | 1,13E+04 | Non-alcoholic fatty liver disease (NAFLD) |
| 68 | 75 | 1,58E+03 | 2,14E+04 | Pancreatic cancer |
| 163 | 215 | 1,26E+04 | 1,64E+05 | Epstein-Barr virus infection |
| 60 | 66 | 1,98E+04 | 2,48E+05 | Mitophagy - animal |
| 97 | 118 | 3,41E+04 | 4,12E+05 | Carbon metabolism |
| 70 | 80 | 3,84E+04 | 4,47E+04 | RNA degradation |
| 105 | 130 | 4,80E+04 | 5,40E+05 | FoxO signaling pathway |
| 135 | 175 | 6,57E+04 | 7,08E+05 | Hepatocellular carcinoma |
| 74 | 86 | 6,74E+04 | 7,08E+05 | ErbB signaling pathway |
| 259 | 369 | 7,33E+04 | 7,47E+05 | Human papillomavirus infection |
| 82 | 98 | 1,48E+05 | 1,42E+06 | Pyrimidine metabolism |
| 53 | 58 | 1,48E+05 | 1,42E+06 | Endometrial cancer |
| 95 | 117 | 1,91E+05 | 1,69E+06 | Oocyte meiosis |
| 79 | 94 | 1,94E+04 | 1,69E+06 | Prostate cancer |
| 116 | 148 | 1,94E+05 | 1,69E+06 | Hepatitis B |
| 120 | 154 | 1,97E+05 | 1,69E+06 | mTOR signaling pathway |
| 61 | 69 | 2,13E+05 | 1,78E+06 | Renal cell carcinoma |
| 78 | 93 | 2,99E+05 | 2,42E+06 | Endocrine resistance |
| 96 | 119 | 3,04E+05 | 2,42E+06 | Sphingolipid signaling pathway |
| 67 | 78 | 5,17E+05 | 4,02E+06 | p53 signaling pathway |
| 151 | 203 | 6,27E+05 | 4,75E+06 | Ribosome |
| 149 | 200 | 6,59E+05 | 4,89E+06 | Proteoglycans in cancer |
| 135 | 179 | 9,02E+05 | 6,53E+06 | Axon guidance |
| 64 | 75 | 2,01E+06 | 1,42E+07 | Inositol phosphate metabolism |
| 105 | 135 | 2,44E+06 | 1,69E+07 | Estrogen signaling pathway |
| 29 | 29 | 2,84E+06 | 1,93E+07 | RNA polymerase |
| 116 | 152 | 3,18E+06 | 2,12E+07 | Apoptosis |
| 115 | 151 | 4,47E+06 | 2,92E+07 | Hippo signaling pathway |
| 171 | 238 | 4,61E+06 | 2,94E+07 | Human cytomegalovirus infection |
| 62 | 73 | 4,91E+06 | 3,08E+07 | Adherens junction |
| 66 | 79 | 6,90E+06 | 4,24E+07 | Platinum drug resistance |
| 72 | 88 | 9,82E+05 | 5,93E+07 | Progesterone-mediated oocyte maturation |
| 91 | 116 | 1,01E+07 | 6,00E+07 | Thyroid hormone signaling pathway |
| 41 | 45 | 1,47E+07 | 8,38E+07 | Proteasome |
| 41 | 45 | 1,47E+07 | 8,38E+07 | Nucleotide excision repair |
| 54 | 63 | 1,94E+07 | 1,08E+08 | Lysine degradation |
| 81 | 102 | 1,96E+06 | 1,08E+08 | Phosphatidylinositol signaling system |
| 72 | 89 | 2,38E+07 | 1,29E+08 | Longevity regulating pathway |
| 67 | 82 | 3,05E+07 | 1,60E+08 | EGFR tyrosine kinase inhibitor resistance |
| 67 | 82 | 3,05E+07 | 1,60E+08 | Peroxisome |
| 25 | 25 | 3,14E+07 | 1,62E+07 | Glycosylphosphatidylinositol (GPI)-anchor biosynthesis |
| 131 | 179 | 3,18E+07 | 1,62E+07 | Purine metabolism |
| 69 | 85 | 3,30E+07 | 1,66E+07 | Ribosome biogenesis in eukaryotes |
| 36 | 39 | 3,94E+07 | 1,95E+08 | Bladder cancer |
| 145 | 202 | 5,35E+07 | 2,60E+08 | Herpes simplex infection |
| 105 | 140 | 6,55E+07 | 3,14E+07 | Insulin signaling pathway |
| 162 | 230 | 8,47E+06 | 4,00E+08 | Human immunodeficiency virus 1 infection |
| 79 | 101 | 8,83E+07 | 4,11E+08 | C-type lectin receptor signaling pathway |
| 30 | 32 | 1,71E+08 | 7,83E+07 | Base excision repair |
| 44 | 51 | 1,88E+08 | 8,40E+08 | N-Glycan biosynthesis |
| 44 | 51 | 1,88E+08 | 8,40E+08 | Fatty acid metabolism |
| 58 | 71 | 1,96E+08 | 8,62E+08 | Glioma |
| 46 | 54 | 2,34E+08 | 1,02E+09 | Fanconi anemia pathway |
| 78 | 101 | 2,58E+07 | 1,10E+09 | HIF-1 signaling pathway |
| 72 | 92 | 2,60E+07 | 1,10E+09 | mRNA surveillance pathway |
| 155 | 222 | 3,35E+08 | 1,40E+09 | Viral carcinogenesis |
| 88 | 117 | 4,30E+08 | 1,77E+09 | TNF signaling pathway |
| 37 | 42 | 4,39E+07 | 1,79E+08 | Homologous recombination |
| 28 | 30 | 5,00E+08 | 2,01E+09 | Citrate cycle (TCA cycle) |
| 68 | 87 | 5,36E+08 | 2,13E+09 | TGF-beta signaling pathway |
| 127 | 179 | 7,18E+08 | 2,82E+09 | Tight junction |
| 75 | 98 | 7,33E+07 | 2,84E+09 | Small cell lung cancer |
| 54 | 67 | 9,72E+08 | 3,73E+09 | Non-small cell lung cancer |
| 85 | 114 | 1,13E+09 | 4,28E+09 | Toxoplasmosis |
| 40 | 47 | 1,16E+09 | 4,33E+09 | Hedgehog signaling pathway |
| 92 | 125 | 1,23E+09 | 4,57E+09 | AMPK signaling pathway |
| 32 | 36 | 1,34E+09 | 4,90E+09 | DNA replication |
| 71 | 93 | 1,49E+09 | 5,40E+09 | GnRH signaling pathway |
| 34 | 39 | 1,83E+09 | 6,55E+09 | Thyroid cancer |
| 148 | 215 | 1,88E+09 | 6,66E+09 | Regulation of actin cytoskeleton |
| 50 | 62 | 2,02E+09 | 7,09E+09 | Longevity regulating pathway - multiple species |
| 41 | 49 | 2,11E+09 | 7,32E+09 | Sphingolipid metabolism |
| 31 | 35 | 2,18E+09 | 7,47E+09 | Apoptosis - multiple species |
| 60 | 77 | 2,28E+09 | 7,75E+09 | Bacterial invasion of epithelial cells |
| 53 | 67 | 3,21E+09 | 0.00010 | Acute myeloid leukemia |
| 30 | 34 | 3,53E+09 | 0.00011 | Autophagy - other |
| 137 | 199 | 3,78E+09 | 0.00012 | Kaposi sarcoma-associated herpesvirus infection |
| 50 | 63 | 4,57E+09 | 0.00014 | Central carbon metabolism in cancer |
| 142 | 208 | 5,29E+09 | 0.00017 | Focal adhesion |
| 104 | 147 | 5,77E+09 | 0.00018 | Wnt signaling pathway |
| 45 | 56 | 6,27E+09 | 0.00019 | VEGF signaling pathway |
| 98 | 138 | 7,40E+09 | 0.00023 | Apelin signaling pathway |
| 140 | 206 | 8,37E+09 | 0.00025 | Rap1 signaling pathway |
| 28 | 32 | 9,20E+09 | 0.00028 | Propanoate metabolism |
| 28 | 32 | 9,20E+09 | 0.00028 | Circadian rhythm |
| 78 | 107 | 9,73E+08 | 0.00029 | Parathyroid hormone synthesis, secretion and action |
| 30 | 35 | 0.00011 | 0.00034 | SNARE interactions in vesicular transport |
| 39 | 48 | 0.00012 | 0.00036 | Notch signaling pathway |
| 75 | 103 | 0.00014 | 0.000411 | Choline metabolism in cancer |
| 73 | 100 | 0.00015 | 0.000433 | Glucagon signaling pathway |
| 102 | 146 | 0.00015 | 0.000433 | Phospholipase D signaling pathway |
| 115 | 167 | 0.00015 | 0.000437 | NOD-like receptor signaling pathway |
| 29 | 34 | 0.00018 | 0.000520 | Fructose and mannose metabolism |
| 21 | 23 | 0.00019 | 0.000558 | Mismatch repair |
| 107 | 155 | 0.00022 | 0.000615 | Cushing syndrome |
| 47 | 61 | 0.00028 | 0.000791 | Glutathione metabolism |
| 41 | 52 | 0.00029 | 0.00079 | Amino sugar and nucleotide sugar metabolism |
| 43 | 55 | 0.00029 | 0.00079 | Valine, leucine and isoleucine degradation |
| 103 | 150 | 0.00039 | 0.00105 | Breast cancer |
| 27 | 32 | 0.00044 | 0.00119 | Glyoxylate and dicarboxylate metabolism |
| 74 | 104 | 0.00050 | 0.00132 | Glycerophospholipid metabolism |
| 51 | 68 | 0.00050 | 0.00132 | B cell receptor signaling pathway |
| 79 | 112 | 0.00050 | 0.00132 | Insulin resistance |
| 92 | 133 | 0.00053 | 0.00138 | Hepatitis C |
| 19 | 21 | 0.00055 | 0.00142 | Terpenoid backbone biosynthesis |
| 56 | 76 | 0.00058 | 0.00150 | Biosynthesis of amino acids |
| 24 | 28 | 0.00059 | 0.00150 | Hippo signaling pathway - multiple species |
| 21 | 24 | 0.00076 | 0.00190 | Protein export |
| 28 | 34 | 0.00077 | 0.00191 | Fatty acid elongation |
| 69 | 97 | 0.00077 | 0.00191 | T cell receptor signaling pathway |
| 36 | 46 | 0.00088 | 0.00215 | Ferroptosis |
| 36 | 46 | 0.00088 | 0.00215 | Vasopressin-regulated water reabsorption |
| 154 | 237 | 0.00099 | 0.00239 | Ras signaling pathway |
| 86 | 125 | 0.00104 | 0.00251 | Osteoclast differentiation |
| 43 | 57 | 0.00113 | 0.00269 | Legionellosis |
| 50 | 68 | 0.00124 | 0.00293 | Long-term potentiation |
| 35 | 45 | 0.00127 | 0.00299 | Basal transcription factors |
| 118 | 178 | 0.00128 | 0.00299 | Influenza A |
| 65 | 92 | 0.00145 | 0.00336 | NF-kappa B signaling pathway |
| 17 | 19 | 0.00151 | 0.00347 | Steroid biosynthesis |
| 38 | 50 | 0.00172 | 0.00392 | Amyotrophic lateral sclerosis (ALS) |
| 19 | 22 | 0.001953 | 0.00442 | Other types of O-glycan biosynthesis |
