# Supplementary material for: Shedding light on cashmere goat hair follicle biology: from morphology analyses to transcriptomic landascape
Source: BMC Genomics. 2020 Jul 2;21:458. doi: 10.1186/s12864-020-06870-x (PMC7330943; doi:10.1186/s12864-020-06870-x)
Supplement: Supplementary file 4 — Additional file 4. Condition and efficiency of primers used for the RT-qPCR experiments. [file 12864_2020_6870_MOESM4_ESM.docx]

**Additional file 4: Condition and efficiency of primers used for the qRT-PCR experiments.**

| **GENE NAME** | **PRIMER 5'-3' (FORWARD & REVERSE)** | **ACCESSION NUMBER** | **AMPLICON LENGHT (BP)** | **EFFICENCY** | **R^2^** |
| --- | --- | --- | --- | --- | --- |
| **HOUSEKEEPING GENES** | |  |  |  |  |
| SDHA | AGCACTGGAGGAAGCACAC CACAGTCGGTCTCGTTCAA | XM_018065656.1 | 105 | 105,5 | 1 |
| UBC | GCATTGTTGGGTTCCTGTGT CTCACAGGTCAAAATGCAAA | XM_005693600.3 | 90 | 85,1 | 0,96 |
| **TARGET GENES** |  |  |  |  |  |
| CP | AGAAATGGAAGTCTTGGAGAGGA CACATTTTCAGGCTCTGTGGTAA | XM_013965432.1 | 141 | 100 | 1 |
| ELOVL3 | GAGCAGATGTTCCAGCCCTA GTTTTGCCCCACGAAGATGA | XM_005698356.2 | 126 | 98,2 | 0,99 |
| K4 | ACAACAAGTTTGCCTCCTTCATC CTCAAGGTTTTTGACAGATGTGG | XM_005679934 | 122 | 103,9 | 0,99 |
| K13 | GTACAAGATGCTGCTGGACA AAGGCCTACTGTTTTCTGGAA | XM_018065053.1 | 158 | 96,3 | 0,97 |
| PLIN4 | CCCAGCTTGAGGATGCCTTC CTTCCTTTGCTTGGCTGCTC | XM_018050877.1 | 81 | 108,8 | 0,99 |
