# Supplementary material for: Shedding light on cashmere goat hair follicle biology: from morphology analyses to transcriptomic landascape
Source: BMC Genomics. 2020 Jul 2;21:458. doi: 10.1186/s12864-020-06870-x (PMC7330943; doi:10.1186/s12864-020-06870-x)
Supplement: Supplementary file 5 — Additional file 5.P-value < <0.005 of a target genes evaluated in skin biopsies through ANOVA test. [file 12864_2020_6870_MOESM5_ESM.docx]

**Additional file 5: P-value <0,005 of a target genes evaluated in skin biopsies through ANOVA test.**

| **Target** | **P-Value** | **R^2^** |
| --- | --- | --- |
| *CP* | 0,00 | 0,72 |
| *ELOVL3* | 0,00 | 0,84 |
| *K4* | 0,00 | 0,92 |
| *K13* | 0,00 | 0,48 |
| *PLIN4* | 0,00 | 0,64 |
